# One potent sponge based on plant-protein-polyphenol assemblies for coagulopathic hemostasis

**DOI:** 10.1016/j.mtbio.2025.102171

**Published:** 2025-08-05

**Authors:** Yu Wang, Xin Li, Hanlu Chen, Yanfen Shi, Yang Li, Guochao Zhang, Yang Hu, Fu-Jian Xu

**Affiliations:** a State Key Laboratory of Chemical Resource Engineering, Key Lab of Biomedical Materials of Natural Macromolecules (Beijing University of Chemical Technology, Ministry of Education), Beijing Laboratory of Biomedical Materials, College of Materials Science and Engineering, Beijing University of Chemical Technology, Beijing, 100029, PR China; b Quzhou Institute for Innovation in Resource Chemical Engineering, Quzhou, 324000, PR China; c Department of Pathology, China-Japan Friendship Hospital, Beijing, 100029, PR China; d Department of General Surgery, China-Japan Friendship Hospital, Beijing, 100029, PR China

**Keywords:** Hemostasis, Sponge, Coating, Zein, Polyphenol

## Abstract

Commercially available gelatin sponges are widely used in coagulation-dependent bleeding wounds due to its porous structure that concentrates blood cells, and coagulation factors. However, effective hemostasis cannot currently be achieved under coagulopathic conditions. In this study, we designed a procoagulant zein-polyphenol conjugate (ZC) nanoassemblies prepared using an anti-solvent strategy, were directly applied to the surface functionalization of commercially available gelatin sponges. Polyphenols give ZC coatings excellent adhesion and enhance the gelatin sponge's procoagulant properties. Leveraging their distinctive secondary structure, the optimized ZC nanoparticle-coated sponges demonstrated enhanced *in vitro* hemostatic properties compared to unmodified commercial gelatin sponges and exhibited superior red blood cell and platelet adhesion characteristics. Additionally, the sponges enhanced with the ZC coating exhibited superior hemostatic potential in a femoral-artery-injury model, under both coagulation-dependent and coagulopathic conditions. The current study presents a promising approach for the utilisation of zein-based procoagulant materials for versatile hemostatic materials engineering applications.

## Graphical abstract

Procoagulant zein-polyphenol conjugate (ZC) nanoassemblies were synthesized via an anti-solvent precipitation strategy for surface functionalization of commercial gelatin sponges, which achieved significantly improved hemostasis under both healthy and coagulopathic conditions.

**Figure:**
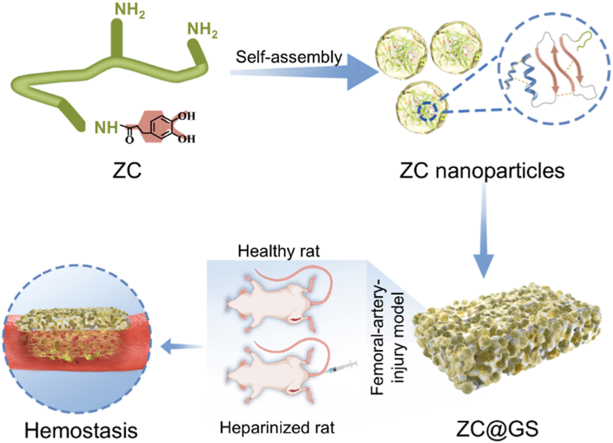

## 1 Introduction

Uncontrolled bleeding on the battlefield, as well as in daily life, can result in various complications, including hemorrhagic shock, coagulopathy, sepsis, and potentially death [1,2]. Consequently, the implementation of interventions to minimize blood loss appears essential. Throughout the years, numerous types of dressings have been developed to halt bleeding, including tourniquets, styptic powders, and hemostatic sponges [3]. Of these, sponges are 3D materials with a porous structure and are characterised by their rapid blood absorption, blood cell concentration, and platelet aggregation properties [[4], [5], [6]]. Gelatin-based sponge (GS) has long been utilised as a local hemostatic agent owing to its superior safety, cost-effectiveness, and ease of use [[7], [8], [9]]. Nevertheless, owing to the absence of an active procoagulant surface, GS and other commercial sponges are incapable of mitigating the risk of haemorrhage.

Functional coatings offer an effective strategy to improve the hemostatic properties of substrates [[10], [11], [12], [13]]. Inspired by mussel biomimetic adhesion, compounds containing phenolic hydroxyl groups offer the ability to adhere to substrates, thus allowing the modification of their surfaces. Within this context, the chemical conjugation of a polyphenol molecule, such as gallic acid, onto amine-rich polymers (i.e., natural chitosan and gelatin), which serve as adhesive catecholamines, has facilitated the development of a series of polymer-polyphenol conjugates [[14], [15], [16], [17], [18]]. Furthermore, these polymer-polyphenol conjugates also exhibit adhesive properties for surface functionalization and harbour hemostatic capabilities [16,19,20].

Recently, zein-based materials have emerged as promising biomaterials due to their amphiphilic characteristics (facilitating diverse protein assemblies), exceptional solubility (high stability in aqueous environments), and the abundance of maize as a source material [[21], [22], [23]]. In our previous study, zein assembly nanoparticles have been reported to exhibit procoagulant properties, including strong adhesion to platelets and red blood cells (RBCs) to accelerate blood clotting rate [24]. However, the use of zein nanoparticles to avoid the risks of intravascular thrombus relies on glutaraldehyde cross-linking between nanoparticles and amino-containing sponges. Nonetheless, given the benefits of polymer-polyphenol conjugates and zein-based materials for hemostatic applications, it is both feasible and beneficial to investigate zein-polyphenol assemblies for the development of functional coatings that can provide novel hemostatic sponges with enhanced procoagulant surfaces.

**Fig. 1:**
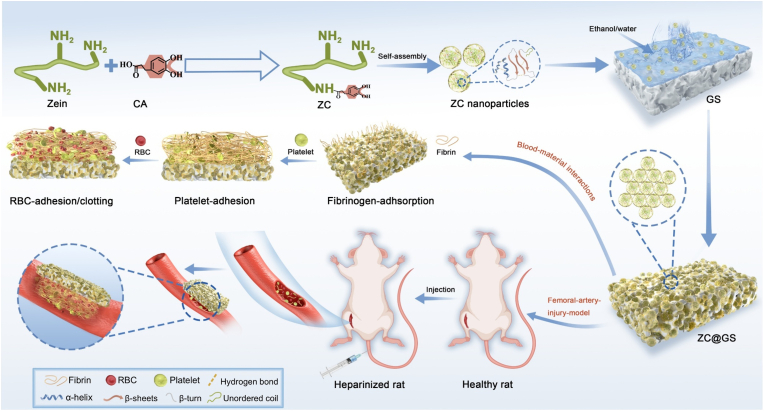
Synthesis route of ZC, schematic illustration of the preparation of Z@GS and ZC@GS sponge for hemostatic applications.

The present study involved the amidation of hydrocaffeic acid, which contains catechol groups, with zein to produce functionally modified products (ZC). The dispersion of ZC nanoassemblies was prepared using an anti-solvent strategy under varying ethanol/water content conditions and was directly utilised for the surface functionalization of commercially available gelatin sponges (ZC@GS). Both cellular and histocompatibility assays were conducted to confirm that the ZC@GS sponge had favorable biocompatibility. The investigation of the *in vitro* hemostasis mechanism elucidated the correlation between the ZC coating's structure and its hemostatic efficacy. Additionally, the *in vivo* hemostatic efficacy of ZC@GS was examined in a healthy rat model for femoral-artery-injury. Considering the superior hemostatic efficacy of the ZC-functionalised coatings *in vitro*, the feasibility for further examination of the hemostatic performance of ZC@GS in a coagulopathic model was also assessed (Fig. 1).

## 2 Experimental section

### 2.1 Materials

Zein (from corn, product no. Z0001) was purchased from TCI Chemical Shanghai (China). 3-(3,4-Dihydroxyphenyl) propionic acid (CA) was purchased from Energy Chemical (China). The absorbable gelatin sponge (GS) was purchased from Jinling Pharmaceutical (China). The detailed information on other biochemical kits/reagents is available in Supporting Information.

### 2.2 Preparation of ZC

ZC was synthesized using EDC/NHS chemistry. In brief, zein (4 g) was dissolved in a 65 % ethanol/water solution (100 mL). EDC (2 g) and NHS (1 g) were gradually added to the solution, and the pH was adjusted using diluted hydrochloric acid (1 M) before introducing hydrocaffeic acid (CA, 4 g). The solution's pH was then maintained at 5.5. The reaction proceeded at 37 °C under a nitrogen atmosphere with continuous stirring for 24 h. The resulting product was purified through membrane dialysis (Mw = 3500 kDa) in a prepared NaCl (pH = 5.5, 0.1 M) solution for 48 h. The dialyzed product was subsequently frozen at −20 °C and freeze-dried to yield the sample, designated as ZC.

### 2.3 Preparation of Z@GS and ZC@GS sponges

Zein (or ZC) nanoassemblies dispersion was prepared using a previously reported anti-solvent method [24]. The detailed information is available in Supporting Information. To prepare Z@GS and ZC@GS, a dipping and freeze-drying technique was employed. The gelatin sponge (GS) was first compressed under a 5 kg weight, after which the nanoparticle dispersion (with a mass 40 times that of the sponge) was dripped onto the sponge surface, allowing it to spread freely. The treated sponges were then freeze-dried at −80 °C to obtain the coated, modified sponges (Z_65_@GS, Z_75_@GS, Z_85_@GS, ZC_65_@GS, ZC_75_@GS and ZC_85_@GS), which was stored at room temperature under vacuum.

### 2.4 Physical characterization

The covalent bonding between zein (Z) and CA was confirmed using proton nuclear magnetic resonance (^1^H NMR) spectroscopy (DMSO, 400 MHz) and fluorescence spectrophotometer (Hitachi F-7000, Japan) [25], while the degree of catechol conjugation was determined through elemental analysis (UNICUBE® trace, Germany). The secondary structures of Z and ZC nanoassemblies were characterized using FTIR spectroscopy [24]. The morphologies of the Z (or ZC) nanoassemblies and sponges (GS, Z@GS and ZC@GS) were examined via SEM (JEOL, JSM-7500F, Japan). Porosity and liquid absorption of the GS, Z@GS, and ZC@GS were assessed using methods adapted from previous studies [26,27]. The compressive properties of the sponges were evaluated at RT using an EZ-LX 50N electronic universal testing machine (Shimadzu, Japan) [28]. The antioxidant activity of the GS, Z@GS and ZC@GS sponges was evaluated using 2,2-diphenyl-1-picrylhydrazyl (DPPH) scavenging, superoxide radical (O_2_·^-^) scavenging and intracellular reactive oxygen scavenging assay [29,30]. The detailed procedures are available in Supporting Information.

### 2.5 In vitro hemolysis and cell viability assays

Fresh whole blood was collected from healthy SD rats and used to obtain RBCs suspensions for the *in vitro* hemolysis assay of GS, Z@GS and ZC@GS. The hemolysis rate and cell viability of GS, Z@GS and ZC@GS were measured using the procedures of hemolysis and standard MTT assay as described in previous work [13,31,32]. The detailed information is available in Supporting Information.

### 2.6 In vitro hemostatic assays

The hemostatic properties of the GS, Z@GS and ZC@GS sponges were evaluated using the blood clotting index (BCI) assay. Interactions between blood components and GS, Z@GS and ZC@GS sponges were assessed through prothrombin time (PT), activated partial thromboplastin time (APTT), and RBCs or platelet adhesion assays. Notably, the RBCs and platelet adhesion assays were conducted in the presence of plasma proteins (PRP). Visualization of adherent RBCs on the GS, Z@GS and ZC@GS sponges was further performed using SEM [26,33]. In addition to whole blood from healthy SD rats, the BCI assay for GS, Z@GS and ZC@GS were also conducted using whole blood freshly collected from heparinized SD rats [24]. The whole-protein-adsorption and Fgn-absorption assays of GS (or Z@GS or ZC@GS) under PPP condition were also performed [34]. The detailed information is available in Supporting Information.

### 2.7 In vivo hemostatic study in rat femoral-artery-injury and liver injury models

The *in vivo* experimental protocols were approved by the Animal Ethics Committee of 10.13039/501100012173China-Japan Friendship Hospital (Beijing, China, grant no. ZDRWLL240004), and all animal procedures were conducted in accordance with the institutional guidelines of the Institute of Clinical Medicine, China-Japan Friendship Hospital. A femoral-artery-injury model was established in healthy SD rats, as described in our previous study [24]. Briefly, the SD rats (male, 160–190 g) were randomly assigned to three groups to evaluate the hemostatic performance of GS, Z_65_@GS and ZC_65_@GS. Following the femoral artery incision, free bleeding was allowed for 15 s before covering the wound with a piece of sponge (2 × 2 cm). Each sponge was weighted down (100 g weight, as standard pressure) for the first 2 min, and bleeding was subsequently monitored every minute until hemostasis was achieved. Blood loss and bleeding time were recorded to assess the hemostatic performance across the three groups. To further evaluate the hemostatic efficacy of the samples, a liver-injury model was established in healthy SD rats. The detailed procedures are available in Supporting Information.

### 2.8 In vitro and *in vivo* degradability and histocompatibility assays

*In vitro degradability study in PBS:* Pre-weighed sponges (m_0_) were immersed in 1 mL of phosphate-buffered saline (PBS, pH = 7.4) and maintained under continuous stirring at 37 °C [35]. At specified incubation or degradation time intervals (1, 3, 5 and 7 d), the sponges were removed, lyophilized, and reweighed to assess changes in mass (m_1_). The weight remaining (%) was calculated from Equation (1),

(1) $$\text{weight}\;\text{remaining}\;\left(\%\right)=\left(1‐\frac{m_{1}}{m_{0}}\right)\times 100\%\text{.}$$

*In vivo degradability study in a rat subcutaneous-implant model:* SD rats were anesthetized with isoflurane, and the hair at the surgical site was shaved. For each rat, an incision (∼2.0 cm in length) was made on the back, and the skin and muscle were separated. GS and ZC_65_@GS sponges (5 mm × 4 mm, pre-sterilized by ultraviolet radiation) were implanted into the incisions, photographed using a digital camera, and the incisions were closed with surgical sutures (day 0). On day 7 and 14, the SD rats were sacrificed, and the incisions were reopened to examine the residual sponges. The blood samples of the GS and ZC_65_@GS-treated rats (with untreated rats as control groups) were evaluated using hematological parameter analysis. Blood samples from SD rats in each group were collected in 2 mL tubes (containing EDTA) for hematological parameter analysis [36,37]. Tissue samples surrounding the sponges, along with major organs (heart, liver, spleen, lung and kidney), were harvested and sectioned for hematoxylin and eosin (H&E) staining.

### 2.9 Statistical analysis

All data were statistically analysed using GraphPad Prism Version 8.3.0 (GraphPad Software, USA). Discrepancies between two groups were analysed by Student's t-tests, and one-way ANOVA was used for discerning discrepancies between multiple groups. In all pictures, asterisk denotes statistically significant differences (∗p < 0.05, ∗∗p < 0.01, ∗∗∗p < 0.001, and ∗∗∗∗p < 0.0001). The quantitative results are expressed using the mean ± SD.

## 3 Results and discussion

### 3.1 Preparation and characterization of ZC

**Fig. 2:**
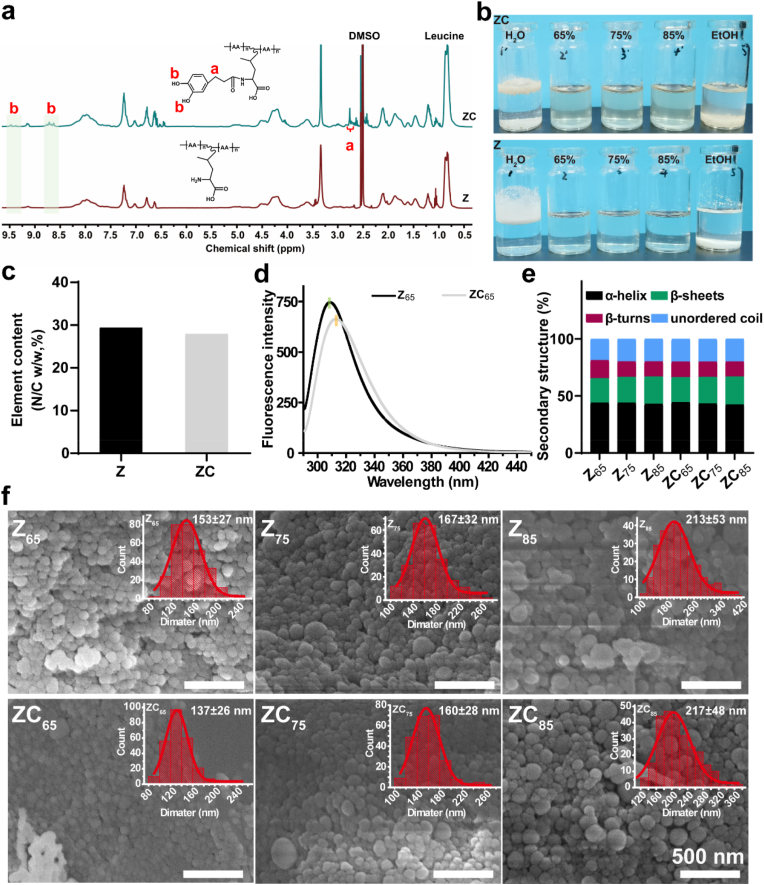
(a) ^1^H NMR spectra, (b) conditions of dissolution in different solvent, (c) elemental analysis results (N/C ratio) and (d) fluorescence spectra of Z and ZC. (e) Secondary structure content by FTIR analysis and (f) SEM images and corresponding size distribution (inset) of Z and ZC nanoassemblies (n ≥ 200).

This present study involved the formation of a zein-polyphenol conjugate (ZC) through the activation of the carboxyl group of CA by EDC/NHS, resulting in the formation of an amide bond with the amino group of Z. The Z and ZC structures were analysed via ^1^H NMR spectroscopy, as illustrated in Fig. 2a. A signal peak at *δ* = 6.6 ppm was observed in the ^1^H NMR spectrum, indicating the presence of the C-H bond of the benzene ring in hydrocaffeic acid, which was not present in unmodified Z. Protons at the α and β positions in CA were detected at *δ* = 2.8 ppm and *δ* = 2.5 ppm, respectively. Furthermore, the protons of the phenolic hydroxyl groups on the catechol moiety were observed at *δ* = 8.7 ppm and *δ* = 9.4 ppm, indicating the successful covalent coupling of CA with Z. As shown in Fig. 2b, owing to the high concentration of non-polar amino acids, Z exhibited no dissolution in deionised water and ethanol. Conversely, Z completely dissolved in 65 %, 75 %, and 85 % ethanol-water solutions, resulting in clear and transparent solutions, corroborating prior research findings [24]. The dissolution of ZC is consistent with that of Z, possibly due to a low degree of substitution, which is conducive to maintaining the self-assembly characteristics of zein. The percentage contents of nitrogen/carbon (N/C) elements in Z and ZC were 29.37 % and 27.91 %, respectively (Fig. 2c, determined by organic element analysis). Accordingly, the degree of catechol conjugation of ZC was calculated to be 6.03 % (Table S1).

Fluorescence spectroscopy evaluates alterations in protein spatial conformation by observing the responsiveness of fluorescent groups to their microenvironment. Aromatic amino acid residues, which are more prevalent in Z, have been documented to emit at a peak wavelength of approximately 304 nm. Alterations in the phenolic environment lead to modified fluorescence spectra, thereby enabling the method to yield insights into structural changes. Fig. 2d displays the fluorescence emission spectra of Z and ZC. When compared to Z_65_, the fluorescence intensity of ZC_65_ grafted with CA was found to be significantly reduced, suggesting that the interaction between the benzene ring of ZC phenolic compounds and aromatic amino acid residues may have obscured the fluorescent chromophores, thus limiting their exposure to surrounding solvents and attenuating the fluorescence. Furthermore, ZC_65_ demonstrated a notable red shift in its maximum emission wavelength (Z_65_: 308.5 nm, ZC_65_: 313.5 nm), which was likely due to the covalent attachment of polyphenols to proteins, resulting in a more flexible protein conformation and increasing the exposure of aromatic amino acid residues to a more hydrophilic environment [25,38]. As observed, the results demonstrate covalent interactions of CA with aromatic amino acid groups, thereby elucidating the successful coupling of CA with Z.

Z and ZC can also perform an anti-solvent strategy in ethanol-water solvents of varying concentrations; the resulting solutions were observed to change from clear to milky white, suggesting the formation of insoluble assemblies (Fig. S1). The corresponding Z and ZC assemblies (Z_65_, Z_75_, Z_85_, ZC_65_, ZC_75_ and ZC_85_) were thus named according to their Z/ZC composition as well as the solvent ratio (v/v, ethanol/water). The Z and ZC assemblies, prepared using an anti-solvent strategy in various ethanol-water solutions, were then analysed through FTIR spectroscopy (Fig. S2 and S3). Additionally, deconvolution and curve fitting of the amide I band spectrum (1600–1700 cm^−1^) were conducted to ascertain the secondary structure composition of Z and ZC assemblies. Fig. 2e illustrates the composition of secondary structures (α-helix, β-sheets, β-turns, and unordered coils) in Z and ZC. The Z_65_ produced via the anti-solvent strategy exhibited a reduced β-sheets content (21.4 %) and an elevated β-turns content (15.8 %) in comparison to Z_75_ and Z_85_. In addition, the β-sheets (21.8 %) of ZC_65_ were lower than those of ZC_75_ and ZC_85_, while the β-turns (13.6 %) were higher than those of ZC_75_ and ZC_85_. The significant change in the secondary structure results from anti-solvent precipitation, which induces intermolecular and intramolecular hydrogen bonds during the self-assembly process into the assemblies [39,40]. Furthermore, ZC_65_ exhibited a greater content of β-sheets and a reduced content of β-turns in comparison to Z_65_. This change in the secondary structure content may be attributed to the protein conformation and aggregation state. As shown in Fig. 2f, the SEM images clearly demonstrates the formation of spherical nanoparticles by Z and ZC, which were obtained via an anti-solvent approach. The statistical data indicate that the average particle size of Z and ZC nanoassemblies is comparable, and both progressively increased during the self-assembly process with the increase in the ethanol concentration. This can be ascribed to the increased hydrophobicity of the molecular chains of Z and ZC, which facilitates the formation and growth of nanoparticles.

### 3.2 Preparation and characterization of Z@GS and ZC@GS

**Fig. 3:**
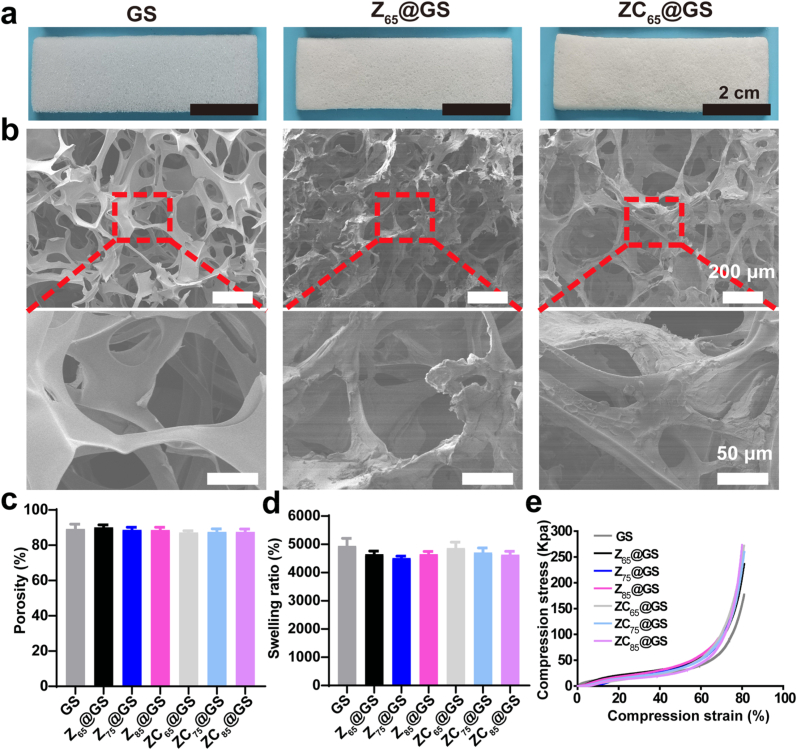
(a) Photograph, (b) SEM images of the top-surface view of GS, Z_65_@GS and ZC_65_@GS. (c) Porosity (data are presented as the mean ± SD, n = 3), (d) **s**welling ratio in PBS at 30 min (data are presented as the mean ± SD, n = 3), and (e) the uniaxial compression stress-strain curves of GS, Z@GS and ZC@GS sponge.

As illustrated in Fig. 1, dispersions of Z and ZC nanoassemblies were employed for the modification of GS via coating, resulting in Z@GS and ZC@GS. Furthermore, as shown in Fig. 3a and Fig. S4a, the unmodified GS was found to be snowy white in appearance, whereas the GS modified with dispersions of Z and ZC nanoassemblies displayed a primrose hue. Subsequently, the surface morphology of GS, Z@GS, and ZC@GS sponges was examined using SEM. As depicted in Fig. 3b and Fig. S4b, the top surface of the GS was smooth in nature, whereas the top surfaces of the Z@GS and ZC@GS sponges exhibited an uneven and rough morphology, indicating the successful application of the Z and ZC coatings on the GS surface. Furthermore, when the surfaces of Z@GS and ZC@GS sponges were examined using SEM at a magnified scale, the coating was observed to constitute a dense layer composed of nanoparticles (Fig. S4c). Thus, it was demonstrated that Z and ZC nanoparticles were effectively coated onto the surface of GS, likely attributable to the non-covalent interactions of proteins and the adhesion properties of catechol groups in CA.

The porosity of GS, Z@GS, and ZC@GS sponges was quantitatively assessed using the ethanol displacement method to further examine the impact of Z and ZC coatings on the pore structure of GS. As shown in Fig. 3c, the porosity of the GS sponge was estimated to be 89 ± 3 %, while the modified sponges Z_65_@GS, Z_75_@GS, Z_85_@GS, ZC_65_@GS, ZC_75_@GS, and ZC_85_@GS exhibited a porosity of ∼89 %. The findings thus indicate that the coating produced by Z and ZC nanoparticles did not influence the three-dimensional pore architecture of GS or the interconnectivity among pore structures. In addition, the liquid-absorption capacity of GS, Z@GS, and ZC@GS sponges was evaluated in phosphate-buffered saline (PBS) after 30 min. As illustrated in Fig. 3d, the liquid absorption capacity of GS in PBS was nearly identical to that of Z@GS and ZC@GS, with all sponges exhibiting a liquid absorption capacity of 45–50 times. The findings thus indicate that Z and ZC coatings did not influence the liquid absorption capacity of the sponge.

To further assess the mechanical properties of the GS, Z@GS, and ZC@GS, the sponges were subjected to the uniaxial compressive test, as illustrated in Fig. 3e. The compressive strength of the GS was estimated to be 177 Kpa at 80 % compressive strain, while the compressive strength of the Z_65_@GS, Z_75_@GS, Z_85_@GS, ZC_65_@GS, ZC_75_@GS, and ZC_85_@GS sponges ranged from 235 to 275 Kpa. This indicates that the sponges can withstand specific pressures that are common in clinical hemostatic applications without readily fracturing, rendering them appropriate for compression hemostasis [41]. Additionally, the stability of the Z_65_ and ZC_65_ coatings on the polyurethane surface in saline was evaluated (Fig. S5). The hydrophobic characteristics of Z assemblies and the catechol groups within the ZC structure guarantee robust adhesion of the ZC coating to the substrate [42].

### 3.3 In vitro hemostatic and antioxidant assays of ZC@GS

**Fig. 4:**
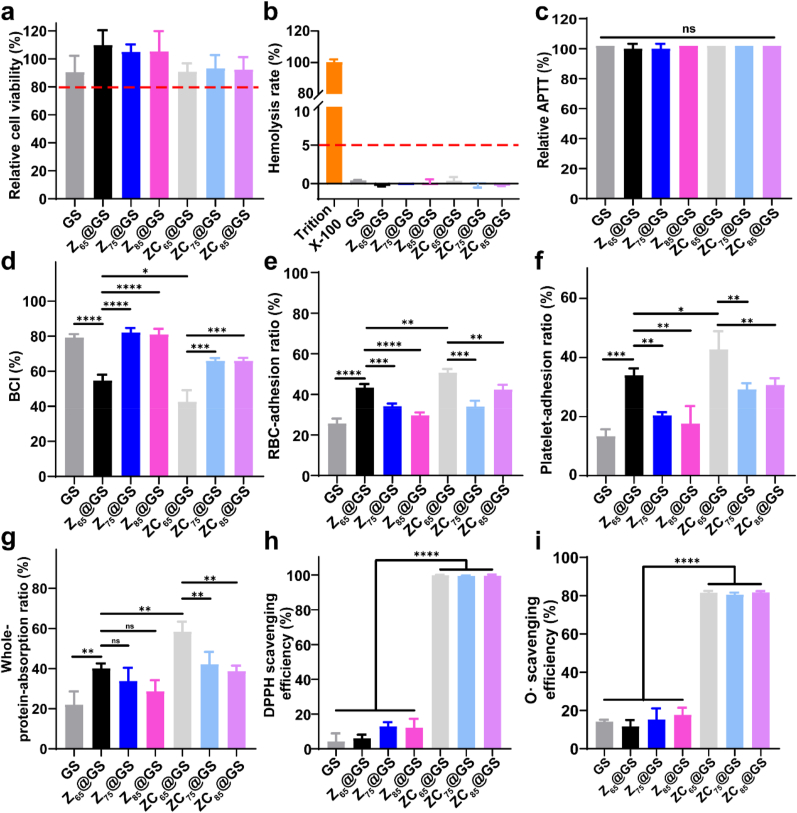
(a) L929 cell viability (data are presented as the mean ± SD, n = 3, one-way ANOVA), (b) hemolysis rate (data are presented as the mean ± SD, n = 3, one-way ANOVA), (c) APTT (data are presented as the mean ± SD, n = 3, one-way ANOVA), (d) BCI value (data are presented as the mean ± SD, n = 3, one-way ANOVA), (e) RBC-adhesion ratio (with plasma proteins) (data are presented as the mean ± SD, n = 3, one-way ANOVA), (f) platelet-adhesion ratio (with plasma proteins) (data are presented as the mean ± SD, n = 3, one-way ANOVA) and (g) whole-protein adhesion ratio under PPP conditions of GS, Z@GS and ZC@GS (data are presented as the mean ± SD, n = 3, one-way ANOVA). The scavenging efficiency of GS, Z@GS and ZC@GS for (h) DPPH free radical (data are presented as the mean ± SD, n = 3, one-way ANOVA) and (i) superoxide free radical (data are presented as the mean ± SD, n = 3, one-way ANOVA). (ns denotes no significant difference, while ∗p < 0.05, ∗∗p < 0.01, and ∗∗∗p < 0.001 represent statistically significant differences, one-way ANOVA).

Cell and blood compatibility testing is essential as a prerequisite for hemostatic applications [32]. Thus, the impact of GS, Z@GS, and ZC@GS extraction on the viability of L929 cells was assessed using the MTT assay (Fig. 4a illustrates the relative cell viability measured by the MTT assay following a 24-h co-culture of the sponge extract with L929 cells). In comparison to GS, both Z@GS and ZC@GS exhibited comparably elevated cell viability, with all measurements exceeding 80 %. Thus, the Z@GS and ZC@GS demonstrated no cytotoxicity and displayed excellent cytocompatibility in the cellular growth milieu. In addition, the state of Z@GS and ZC@GS-treated RBCs was identical to that of the negative control group (clear and transparent, Fig. S6a), indicating that the integrity of the RBCs membrane in the Z@GS and ZC@GS groups was akin to that of the negative control group. Moreover, quantitative data indicated that GS, Z@GS, and ZC@GS exhibited low haemolysis rates (<5 %, Fig. 4b), which was within the permissible range for biological materials. Subsequently, APTT and PT were employed to investigate the impact on the endogenous/exogenous coagulation system (i.e., plasma coagulation). The APTT (Fig. 4c) and PT (Fig. S6b) of the Z@GS (including Z_65_@GS, Z_75_@GS and Z_85_@GS) and ZC@GS (including ZC_65_@GS, ZC_75_@GS and ZC_85_@GS) groups were comparable to the corresponding control group (untreated plasma) and remained within the normal test range, indicating that the plasma-involved coagulation system was neither inhibited nor enhanced following exposure to Z@GS and ZC@GS. These results, thus, demonstrate that Z@GS and ZC@GS exhibit excellent cytocompatibility and blood compatibility, indicating significant potential as hemostatic materials.

The *in vitro* hemostatic efficacy of GS, Z@GS, and ZC@GS was further assessed using the blood-clotting index (BCI) assay. As shown in Fig. 4d, Z_65_@GS exhibited lower BCI values compared to GS, Z_75_@GS, and Z_85_@GS. This was potentially attributable to the improved hemostatic properties of Z_65_ following assembly, which was characterised by a distinct secondary structure. More importantly, ZC_65_@GS exhibited lower BCI values compared to Z_65_@GS, ZC_75_@GS, ZC_85_@GS, and GS, demonstrating the most optimal *in vitro* hemostatic performance among all sponges. Besides, the dynamic BCI values of ZC_65_@GS were also lower than GS and Z_65_@GS at the same time interval (Fig. S7). Phenolic hydroxyl groups of CA present in ZC can bind to cells and proteins [19]. As shown in Fig. 4e–g and Fig. S8-9, the adhesion of RBCs, platelets, plasma proteins and Fgn to ZC_65_@GS was markedly higher in comparison to that to Z_65_@GS and GS. Given that the enhancement of blood cell/plasma protein binding by polyphenolic hydroxyl is widely acknowledged as advantageous for blood clot formation, the findings indicate the superior nature of ZC_65_@GS [43,44]. On the other hand, the adhesion ratios of RBCs, platelets, and plasma proteins were significantly reduced in Z_75_@GS and Z_85_@GS compared to Z_65_@GS (Fig. 4e–g), with the corresponding results also being significantly lower in Z_75_@GS and Z_85_@GS than in Z_65_@GS.

Wound dressings exhibiting antioxidant properties have the ability to regulate the excessive generation of reactive oxygen species (ROS), thereby promoting wound healing and diminishing tissue inflammation. Herein, the antioxidant capacity of GS, Z@GS, and ZC@GS sponges was evaluated by quantifying their DPPH and superoxide radical scavenging abilities. As illustrated in Fig. 4h and i, the DPPH radical scavenging capacity and superoxide radical scavenging capacity of GS and Z@GS were found to be below 20 %. Conversely, the DPPH and superoxide radical scavenging capacities of the ZC@GS sponges were markedly superior to those of the Z@GS sponge, with scavenging efficiencies exceeding 80 % in all cases. The *in vitro* antioxidant property of ZC@GS were further assessed by the intracellular reactive oxygen species (ROS) scavenging assay (Fig. S10). ZC@GS exhibited significantly enhanced ROS scavenging efficiency, reaching as high as 90 %, compared to both the Z@GS and GS samples. All these results demonstrated the favorable antioxidant properties of ZC@GS endowed by polyphenol-involved ZC coating.

### 3.4 In vivo assays of ZC@GS for coagulation-dependent/coagulopathic hemostasis

**Fig. 5:**
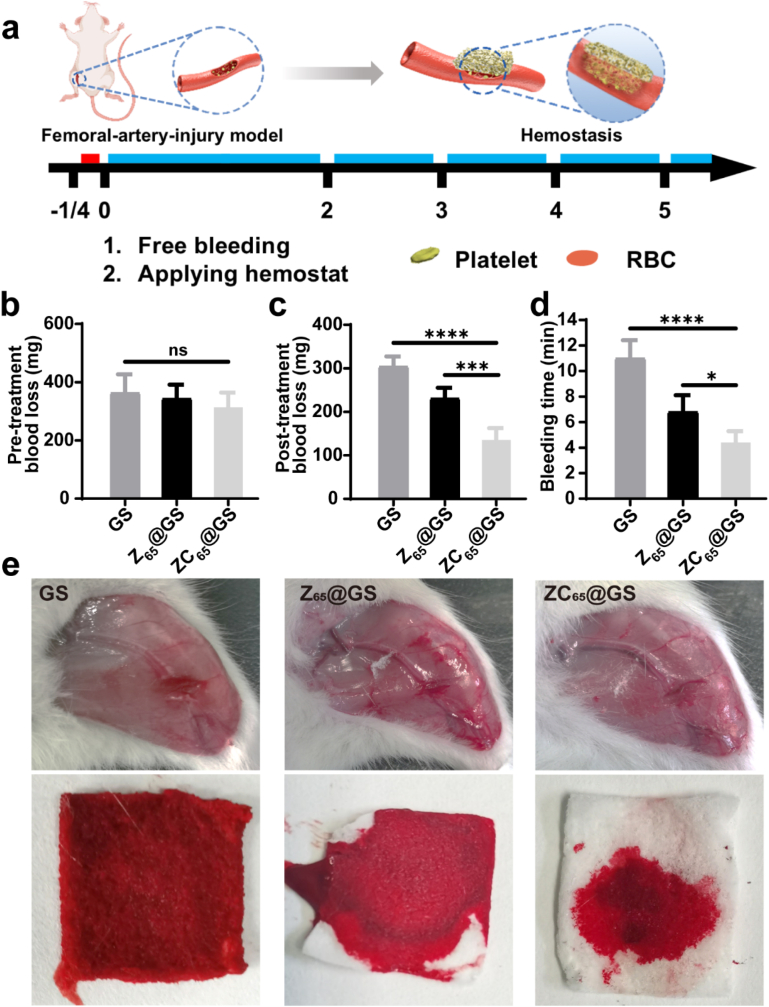
(a) Schematic illustration of the application, (b) pre-treatment blood loss (data are presented as the mean ± SD, n ≥ 3, one-way ANOVA), (c) post-treatment blood loss (data are presented as the mean ± SD, n ≥ 3, one-way ANOVA), (d) bleeding time (data are presented as the mean ± SD, n ≥ 3, one-way ANOVA) and (e) representative photographs of GS, Z_65_@GS and ZC_65_@GS in a rat femoral-artery-injury model. (ns denotes no significant difference, while ∗p < 0.05, ∗∗p < 0.01, and ∗∗∗p < 0.001 represent statistically significant differences, one-way ANOVA).

A femoral-artery-injury model in healthy SD rats was established to evaluate the hemostatic effectiveness of ZC_65_@GS during acute haemorrhage scenarios (with GS and Z_65_@GS as control groups). As depicted in Fig. 5a, to minimize or eliminate the impact of variations in experimental samples, blood loss during the initial 15 s of free bleeding after femoral artery incision was measured using filter paper for collection. Valid samples were selected within the range of 200–450 mg of pre-treatment blood loss, ensuring no significant differences among the three groups in baseline blood loss (Fig. 5b). As shown in Fig. 5c, the post-treatment blood loss following treatment with GS, Z_65_@GS and ZC_65_@GS were 304.3 ± 20.5, 135.3 ± 24.5, and 230.6 ± 22.1 mg, respectively. Post-treatment blood loss in the Z_65_@GS and ZC_65_@GS group were significantly lower compared to the GS group. Furthermore, the bleeding time for GS, Z_65_@GS, and ZC_65_@GS were 11.0 ± 1.3, 6.8 ± 1.2, and 4.4 ± 0.8 min, respectively (Fig. 5d), and the hemostasis times for both Z_65_@GS and ZC_65_@GS were significantly shorter than those for the GS group, while the post-treatment blood losses and wound of the three groups were also visualized by photographs of the three sponges after hemostasis (Fig. 5e).

To further evaluate the hemostatic efficacy of the samples, we established a liver-injury model of rat for irregular or organ-puncture wounds. As shown in Fig. S11a, we measured the blood loss during the initial 10 s of uncontrolled bleeding post-injury to minimize variability between experimental samples. Valid samples were selected based on a pre-treatment blood loss range of 50–150 mg, confirming no significant differences in baseline blood loss among the three groups. As shown in Fig. S11b, the post-treatment blood loss of GS, Z_65_@GS and ZC_65_@GS were 314.3 ± 20.8, 199.7 ± 34.1, and 87 ± 23.5 mg, demonstrates that both the Z_65_@GS and ZC_65_@GS groups exhibited significantly reduced post-treatment blood loss compared to the GS group. Furthermore, the hemostasis times for Z_65_@GS and ZC_65_@GS were significantly shorter than that of GS (Fig. S11c). Photographs of the sponges after hemostasis visually document the differences in post-treatment blood loss among the three groups (Fig. S11d).

**Fig. 6:**
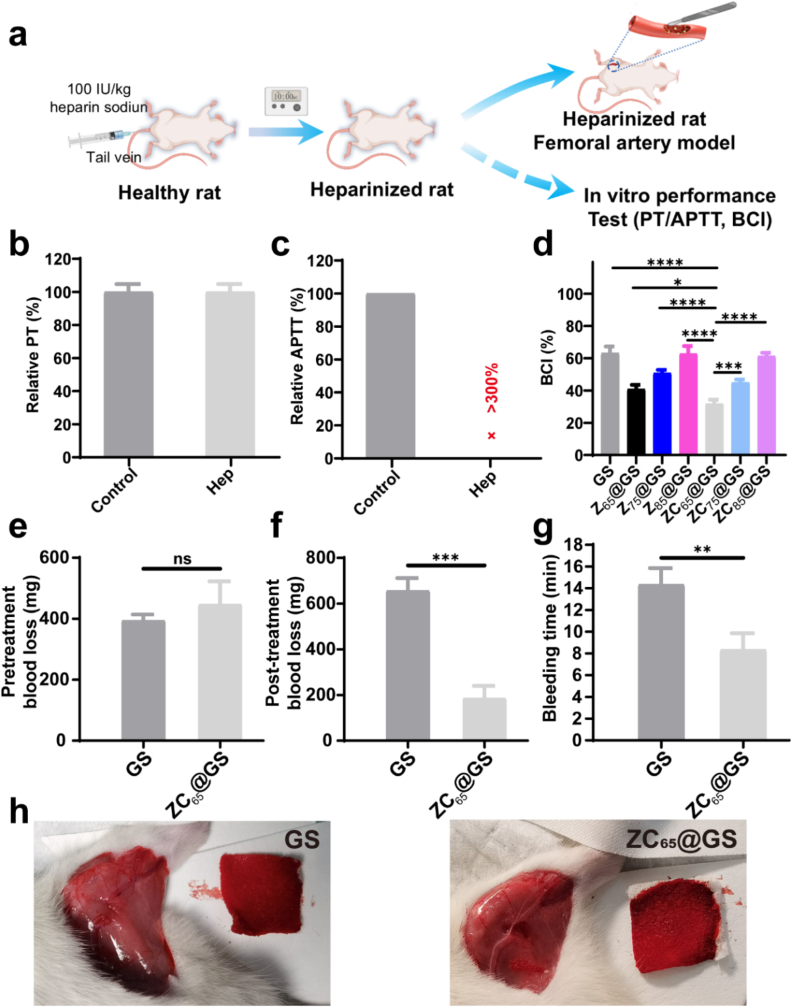
(a) Schematic illustration of the establishment of heparinized rats and corresponding *in vitro*/*in vivo* assays. (b) PT (data are presented as the mean ± SD, n = 3), (c) APTT values of whole blood drawn from heparinized SD rats (data are presented as the mean ± SD, n = 3). (d) BCI value of GS, Z@GS and ZC@GS adopting heparinized blood (data are presented as the mean ± SD, n = 3, one-way ANOVA). (e) Pre-treatment blood loss (data are presented as the mean ± SD, n = 3, Student's t-tests), (f) post-treatment blood loss (data are presented as the mean ± SD, n = 3, Student's t-tests), (g) bleeding time (data are presented as the mean ± SD, n = 3, Student's t-tests) and (h) representative photographs of GS and ZC@GS groups in femoral-artery-injury model of heparinized SD rats. (ns denotes no significant difference, while ∗ p < 0.05, ∗∗p < 0.01, and ∗∗∗p < 0.001 represent statistically significant differences).

Patients administered heparin face an elevated risk of uncontrolled haemorrhage during surgical procedures, complicating effective hemostasis. Particularly, zein-based gel has recently been developed for hemostatic materials owing to the high adhesion of released zein with blood components [22]. Given the remarkable procoagulant characteristics of ZC_65_@GS *in vitro* (RBC/platelet adhesion capability) and its effective hemostatic performance in a femoral-artery-injury model on healthy SD rats, the *in vivo* hemostatic properties of ZC_65_@GS were further assessed by establishing a femoral-artery-injury model in heparinised SD rats [24]. As shown in Fig. 6a, the SD rats were administered heparin (100 IU/kg) via the tail vein to create a coagulopathic model, with hemostatic properties assessed 10 min post-injection. Subsequently, PT and APTT assays were conducted using PPP obtained from the heparinised SD rats. As shown in Fig. 6b, the coagulopathic SD rats exhibited comparable PT values to the healthy SD rats; however, the coagulopathic SD rats demonstrated a markedly prolonged APTT (Fig. 6c), thereby confirming the successful establishment of the coagulation model. In addition, Fig. 6d illustrates that GS had a BCI value of 63.2 ± 3.4 %, indicating GS's inadequate hemostatic capability in anticoagulated whole blood. The BCI values for Z_65_@GS and ZC_65_@GS were 40.9 ± 2.1 % and 31.8 ± 2.1 %, respectively. As observed, both Z_65_@GS and ZC_65_@GS exhibited significantly lower BCI values compared to the GS group, thus demonstrating superior hemostatic performance in anticoagulated whole blood, with ZC_65_@GS exhibiting the most effective hemostatic capability *in vitro*. Additionally, the hemostatic capacity of ZC_65_@GS surpassed that of GS sponges in instances of coagulopathic bleeding, indicating that ZC_65_@GS sponges were anticipated to attain effective hemostasis in the coagulopathic model. In the context of their *in vivo* performance (Fig. 6e–g), the ZC_65_@GS exhibited comparable pretreatment blood loss of 350–550 mg, yet demonstrated a shorter bleeding time compared to GS (14.3 min vs. 8.3 min) and a markedly reduced post-treatment blood loss (657 mg vs. 186 mg). Photographs of sponges after hemostasis clearly show that the amount of post-treatment blood losses in the GS group was significantly more than that in the ZC@GS group. (Fig. 6h). On the one hand, the post-treatment blood loss in the ZC_65_@GS and GS groups of heparinised SD rats were significantly higher than that in the healthy model (Fig. 5b–d), confirming the detrimental impact of the SD rats' coagulopathic condition on hemostasis. On the other hand, the aforementioned results demonstrated the superior hemostatic efficacy of ZC_65_@GS compared to GS in a coagulopathic haemorrhage scenario, suggesting that ZC_65_@GS also achieves effective coagulopathic hemostasis, which is owing to the favorable ZC coating and would be a great advantage than previously reported zein-based sponge [21]. This likely results from the ZC_65_@GS's superior RBCs and plasma protein adhesion properties, along with the sponge's capacity to absorb specific liquids and provide a physical barrier, thereby accelerating blood coagulation and facilitating hemostasis.

### 3.5 In vitro/*in vivo* degradability and histocompatibility assays of ZC@GS

**Fig. 7:**
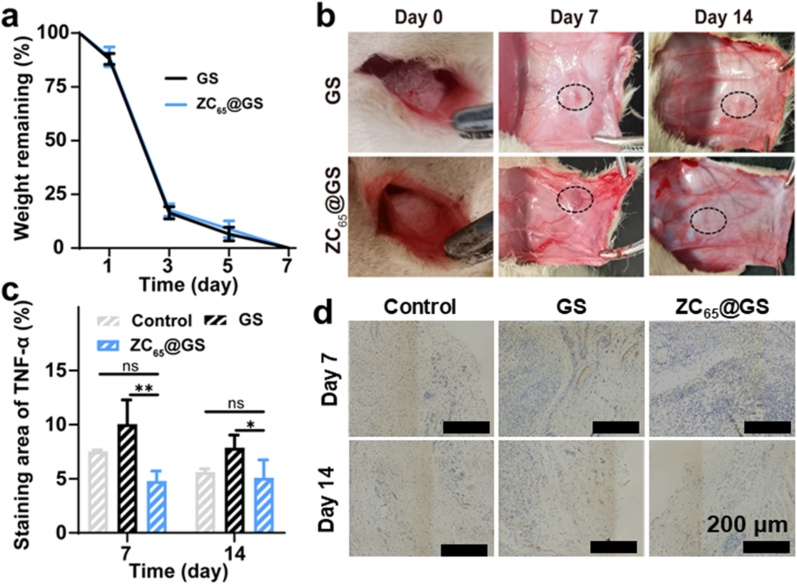
(a) *In vitro* degradation rate (data are presented as the mean ± SD, n = 3), (b) *in vivo* photographs of degradation of GS and ZC_65_@GS (n = 3). (c) Quantitative analysis of positive regions (data are presented as the mean ± SD, n = 3, one-way ANOVA), (d) staining images of tissues surrounding of control, GS and ZC_65_@GS for TNF-α in a subcutaneous implantation model of healthy rats (n = 3). (ns denotes no significant difference, while ∗ p < 0.05, ∗∗p < 0.01, and ∗∗∗p < 0.001 represent statistically significant differences).

Hemostatic materials with good biodegradability are conducive to their application in biological organisms. Herein, the *in vitro* degradability of GS and ZC_65_@GS was evaluated as illustrated in Fig. 7a. Both GS and ZC_65_@GS demonstrated good *in vitro* degradability. To further explore the degradability of the GS and ZC_65_@GS *in vivo*, a GS (or ZC_65_@GS) was subcutaneously implanted in SD rats. As shown in Fig. 7b, the sponges exhibited partial degradability by day 7, with a reduction in volume, and were largely unobservable by day 14, indicating that GS and ZC_65_@GS possess commendable biodegradability.

To assess the histocompatibility of the implanted foreign body, the expression of inflammatory cytokines (TNF-α) in the tissue surrounding the implanted area was also analysed to evaluate the inflammation levels on day 7 and 14 (Fig. 7c and d). As shown in Fig. 7c, TNF-α expression was elevated on day 7 in the GS group relative to the blank control group, whereas TNF-α expression was markedly reduced on day 7 in the ZC_65_@GS group. The statistical findings reveal that during the initial phase, implantation in the GS group results in a higher TNF-α expression in the surrounding tissue. Conversely, the TNF-α expression in the ZC_65_@GS group is reduced, which may be attributed to the superior antioxidant properties of the phenolic hydroxyl groups in ZC_65_@GS. Subsequent examination of inflammation in the adjacent tissues of the sponge via H&E staining revealed that, in contrast to the control group GS, no notable inflammatory cells were detected in ZC_65_@GS (Fig. S12), suggesting that ZC_65_@GS had favorable tissue compatibility. In addition to the above, the blood biochemical parameters, including leukocyte, RBC, platelet, and lymphocyte counts, of SD rats treated with GS and ZC_65_@GS were assayed. The analysis of blood samples on day 7 and 14, as depicted in Fig. S13, revealed no significant differences between SD rats implanted with GS and ZC_65_@GS and healthy SD rats, with all indices remaining within the normal range. Additionally, the principal organs of the SD rats were subjected to staining, and no notable inflammatory responses were detected in any rat, irrespective of the treatment duration (Fig. S13 e and j). Consequently, we determined that GS and ZC_65_@GS can be utilised safely for the treatment of biological trauma without negative effects.

## 4 Conclusions

In this study, hydrocaffeic acid containing catechol groups was chemically coupled to synthesize ZC, followed by the preparation of ZC nanoassemblies dispersion using an antisolvent strategy for the surface modification of GS. *In vitro* cell compatibility and *in vivo* tissue compatibility studies confirmed that ZC@GS exhibited no adverse effects on cells or the organism, demonstrating excellent biocompatibility. The ZC_65_ nanoassemblies, optimized for secondary structure, enhanced the GS (ZC_65_@GS) by promoting superior platelet, RBCs, and plasma protein adhesion, resulting in outstanding *in vitro* hemostatic properties. Furthermore, ZC_65_@GS showed strong antioxidant activity, which helped mitigate tissue inflammation. Building on its impressive *in vitro* procoagulant capabilities, ZC_65_@GS was tested in a simulated acute haemorrhage femoral artery model (healthy and heparinized SD rats), where it significantly reduced blood loss and achieved rapid hemostasis in a shorter time compared to GS alone, demonstrating remarkable hemostatic efficacy. This straightforward coating design for procoagulant assemblies offers considerable promise for broad clinical use, particularly in serving the growing population of patients with coagulopathic disorders.

## CRediT authorship contribution statement

**Yu Wang:** Writing – original draft, Methodology, Investigation, Data curation, Conceptualization. **Xin Li:** Writing – original draft, Methodology, Investigation, Data curation, Conceptualization. **Hanlu Chen:** Methodology, Investigation, Data curation. **Yanfen Shi:** Methodology, Investigation. **Yang Li:** Writing – review & editing, Data curation. **Guochao Zhang:** Writing – review & editing, Supervision, Methodology, Investigation, Funding acquisition, Data curation, Conceptualization. **Yang Hu:** Writing – review & editing, Writing – original draft, Supervision, Methodology, Investigation, Funding acquisition, Data curation, Conceptualization. **Fu-Jian Xu:** Writing – review & editing, Supervision, Funding acquisition, Conceptualization.

## Declaration of competing interest

The authors declare that they have no known competing financial interests or personal relationships that could have appeared to influence the work reported in this paper.

## Data Availability

No data was used for the research described in the article.

## References

[1] Fang Y., Zhang L., Chen Y., Wu S., Weng Y., Liu H. (2023). Polysaccharides based rapid self-crosslinking and wet tissue adhesive hemostatic powders for effective hemostasis. Carbohydr. Polym..

[2] Shuo T., Haoting N., Yuqing W., Liuyun J., Xiang H. (2024). A natural carboxylated sisal fiber/chitosan/kaolin porous sponge for rapid and effective hemostasis. Int. J. Biol. Macromol..

[3] Yang J., Wang T., Zhang L., Fan P., Zhao J., Zheng X., Lai Y., Liu H., Wang S. (2024). Injectable hemostatic hydrogel adhesive with antioxidant, antibacterial and procoagulant properties for hemorrhage wound management. J. Colloid Interface Sci..

[4] Yang X., Liu W., Shi Y., Xi G., Wang M., Liang B., Feng Y., Ren X., Shi C. (2019). Peptide-immobilized starch/PEG sponge with rapid shape recovery and dual-function for both uncontrolled and noncompressible hemorrhage. Acta Biomater..

[5] Zhang Z., Kuang G., Zong S., Liu S., Xiao H., Chen X., Zhou D., Huang Y. (2018). Sandwich-like fibers/sponge composite combining chemotherapy and hemostasis for efficient postoperative prevention of tumor recurrence and metastasis. Adv. Mater..

[6] Liu J.Y., Li Y., Hu Y., Cheng G., Ye E., Shen C., Xu F.-J. (2018). Hemostatic porous sponges of cross-linked hyaluronic acid/cationized dextran by one self-foaming process. Mater. Sci. Eng. C.

[7] Zhu Y.H., Zhou C.Y., Peng X., Wang W., Liu Z., Xie R., Pan D.W., Ju X.J., Chu L.Y. (2024). Dialdehyde starch cross-linked aminated gelatin sponges with excellent hemostatic performance and biocompatibility. Carbohydr. Polym..

[8] Xie X., Li D., Chen Y., Shen Y., Yu F., Wang W., Yuan Z., Morsi Y., Wu J., Mo X. (2021). Conjugate electrospun 3D gelatin nanofiber sponge for rapid hemostasis. Adv. Healthcare Mater..

[9] Huang Y., Bai L., Yang Y., Yin Z., Guo B. (2022). Biodegradable gelatin/silver nanoparticle composite cryogel with excellent antibacterial and antibiofilm activity and hemostasis for Pseudomonas aeruginosa-infected burn wound healing. J. Colloid Interface Sci..

[10] Hwang J., Im P., Kim M.K., Kim J. (2024). Polydopamine-coated silk fiber with controllable length for enhanced hemostatic application. Biomacromolecules.

[11] Zhang X., Liu H., Geng H., Sekhar K.P.C., Song A., Hao J., Cui J. (2023). Biologically derived nanoarchitectonic coatings for the engineering of hemostatic needles. Biomacromolecules.

[12] Liu C., Liu C., Shi Z., Lu W., Liu Z., Liu S., Wang X., Wang X., Huang F. (2023). Sprayable surface-adaptive biocompatible membranes for efficient hemostasis via assembly of chitosan and polyphosphate. Carbohydr. Polym..

[13] Long L., Fan Y., Yang X., Ding X., Hu Y., Zhang G., Xu F.-J. (2022). A hydrophobic cationic polyphenol coating for versatile antibacterial and hemostatic devices. Chem. Eng. J..

[14] Ju J., Jin S., Kim S., Choi J.H., Lee H.A., Son D., Lee H., Shin M. (2022). Addressing the shortcomings of polyphenol-derived adhesives: achievement of long shelf life for effective hemostasis. ACS Appl. Mater. Interfaces.

[15] Ghadban A., Ahmed A.S., Ping Y., Ramos R., Arfin N., Cantaert B., Ramanujan R.V., Miserez A. (2016). Bioinspired pH and magnetic responsive catechol-functionalized chitosan hydrogels with tunable elastic properties. Chem. Commun..

[16] Ryu J.H., Hong S., Lee H. (2015). Bio-inspired adhesive catechol-conjugated chitosan for biomedical applications: a mini review. Acta Biomater..

[17] Ryu J.H., Lee Y., Kong W.H., Kim T.G., Park T.G., Lee H. (2011). Catechol-functionalized chitosan/pluronic hydrogels for tissue adhesives and hemostatic materials. Biomacromolecules.

[18] Kim K., Ryu J.H., Koh M.Y., Yun S.P., Kim S., Park J.P., Jung C.W., Lee M.S., Seo H.I., Kim J.H., Lee H. (2021). Coagulopathy-independent, bioinspired hemostatic materials: a full research story from preclinical models to a human clinical trial. Sci. Adv..

[19] Shin M., Ryu J.H., Kim K., Kim M.J., Jo S., Lee M.S., Lee D.Y., Lee H. (2018). Hemostatic swabs containing polydopamine-like catecholamine chitosan-catechol for normal and coagulopathic animal models. ACS Biomater. Sci. Eng..

[20] Shin M., Park S.G., Oh B.C., Kim K., Jo S., Lee M.S., Oh S.S., Hong S.H., Shin E.C., Kim K.S., Kang S.W., Lee H. (2017). Complete prevention of blood loss with self-sealing haemostatic needles. Nat. Mater..

[21] Zhang Y.-B., Wang H.J., Raza A., Liu C., Yu J., Wang J.Y. (2022). Preparation and evaluation of chitosan/polyvinylpyrrolidone/zein composite hemostatic sponges. Int. J. Biol. Macromol..

[22] Raza A., Zhang Y., Hayat U., Liu C., Song J.-L., Shen N., Chao Y., Wang H.J., Wang J.Y. (2023). Injectable zein gel with in situ self-assembly as hemostatic material. Biomater. Adv..

[23] Wang Y., Ying M., Zhang M., Ren X., Kim I.S. (2021). Development of antibacterial and hemostatic PCL/Zein/ZnO-quaternary ammonium salts NPs composite mats as wound dressings. Macromol. Mater. Eng..

[24] Wang Y., Lin J., Fu H., Yu B., Zhang G., Hu Y., Xu F.-J. (2024). A janus gelatin sponge with a procoagulant nanoparticle-embedded surface for coagulopathic hemostasis. ACS Appl. Mater. Interfaces.

[25] Yan S., Xu J., Zhang S., Li Y. (2021). Effects of flexibility and surface hydrophobicity on emulsifying properties: ultrasound-treated soybean protein isolate. LWT--Food Sci. Technol..

[26] Jimenez-Martin J., Las Heras K., Etxabide A., Uranga J., de la Caba K., Guerrero P., Igartua M., Santos-Vizcaino E., Hernandez R.M. (2022). Green hemostatic sponge-like scaffold composed of soy protein and chitin for the treatment of epistaxis. Mater. Today Bio.

[27] Zhao T., Ren R., Qiao S., Tang X., Chi Z., Jiang F., Liu C. (2025). Multi-crosslinking nanoclay/oxidized cellulose hydrogel bandage with robust mechanical strength, antibacterial and adhesive properties for emergency hemostasis. J. Colloid Interface Sci..

[28] Ding C., Cheng K., Wang Y., Yi Y., Chen X., Li J., Liang K., Zhang M. (2024). Dual green hemostatic sponges constructed by collagen fibers disintegrated from Halocynthia roretzi by a shortcut method. Mater. Today Bio.

[29] Liu J., Yu J., Chen H., Zou Y., Wang Y., Zhou C., Tong L., Wang P., Liu T., Liang J., Sun Y., Zhang X., Fan Y. (2024). Porous gradient hydrogel promotes skin regeneration by angiogenesis. J. Colloid Interface Sci..

[30] Zeng Q., Han K., Zheng C., Bai Q., Wu W., Zhu C., Zhang Y., Cui N., Lu T. (2022). Degradable and self-luminescence porous silicon particles as tissue adhesive for wound closure, monitoring and accelerating wound healing. J. Colloid Interface Sci..

[31] Liu J.Y., Hu Y., Li L., Wang C., Wang J., Li Y., Chen D., Ding X., Shen C., Xu F.-J. (2020). Biomass-derived multilayer-structured microparticles for accelerated hemostasis and bone repair. Adv. Sci..

[32] Su Y., Niu M., Xu K., Xu C., Yang P., Hu Y., Xu F.-J. (2024). Cationic starch microparticles with integrated antibacterial and hemostatic performance. Sci. China Technol. Sci..

[33] Wang X., Yuan K., Su Y., Li X., Meng L., Zhao N., Hu Y., Duan F., Xu F.-J. (2024). Tuning blood–material interactions to generate versatile hemostatic powders and gels. Adv. Healthcare Mater..

[34] Su Y., Chen H., Liu Q., Ding X., Lian R., Hu Y., Xu F.-J. (2024). Thermoresponsive gels with embedded starch microspheres for optimized antibacterial and hemostatic properties. ACS Appl. Mater. Interfaces.

[35] Liu L., Liu L., Chen L., Chen G., Wei Y., Hong F.F. (2024). Synthesis of hemostatic aerogel of TEMPO-oxidized cellulose nanofibers/collagen/chitosan and in vivo/vitro evaluation. Mater. Today Bio.

[36] Jacob Filho W., Lima C.C., Paunksnis M.R.R., Silva A.A., Perilhão M.S., Caldeira M., Bocalini D., de Souza R.R. (2018). Reference database of hematological parameters for growing and aging rats. Aging Male.

[37] Wiedmeyer C.E., Ruben D., Franklin C. (2007). Complete blood count, clinical chemistry, and serology profile by using a single tube of whole blood from mice. J. Am. Assoc. Lab. Anim. Sci..

[38] Yan S., Yao Y., Xie X., Zhang S., Huang Y., Zhu H., Li Y., Qi B. (2022). Comparison of the physical stabilities and oxidation of lipids and proteins in natural and polyphenol-modified soybean protein isolate-stabilized emulsions. Food Res. Int..

[39] Mattice K.D., Marangoni A.G. (2020). Functionalizing zein through antisolvent precipitation from ethanol or aetic acid. Food Chem..

[40] Wang Q., Tang Y., Yang Y., Lei L., Lei X., Zhao J., Zhang Y., Li L., Wang Q., Ming J. (2022). Interactions and structural properties of zein/ferulic acid: the effect of calcium chloride. Food Chem..

[41] Huang Z., Chen H., Wang Y., Xiao T., Guo T., Ren Z., Wu C., Wang Y. (2024). Collagen/curdlan composite sponge for rapid hemostasis and skin wound healing. Int. J. Biol. Macromol..

[42] Gonçalves J., Torres N., Silva S., Gonçalves F., Noro J., Cavaco-Paulo A., Ribeiro A., Silva C. (2020). Zein impart hydrophobic and antimicrobial properties to cotton textiles. React. Funct. Polym..

[43] Liu C., Yao W., Tian M., Wei J., Song Q., Qiao W. (2018). Mussel-inspired degradable antibacterial polydopamine/silica nanoparticle for rapid hemostasis. Biomaterials.

[44] Zhao X., Liang Y., Guo B., Yin Z., Zhu D., Han Y. (2021). Injectable dry cryogels with excellent blood-sucking expansion and blood clotting to cease hemorrhage for lethal deep-wounds, coagulopathy and tissue regeneration. Chem. Eng. J..

