# Supplementary material for: One potent sponge based on plant-protein-polyphenol assemblies for coagulopathic hemostasis
Source: Mater Today Bio. 2025 Aug 5;34:102171. doi: 10.1016/j.mtbio.2025.102171 (PMC12345339; doi:10.1016/j.mtbio.2025.102171)
Supplement: Multimedia component 1 [file mmc1.docx]

**Supplementing Information**

of

**One potent sponge based on plant-protein-polyphenol assemblies for coagulopathic hemostasis**

Yu Wang,^a,#^ Xin Li,^a,b,#^ Hanlu Chen,^a^ Yanfen Shi,^c^ Yang Li,^a^ Guochao Zhang,^d,*^ Yang Hu,^a,b,*^ Fu-Jian Xu^a,*^

^a^State Key Laboratory of Chemical Resource Engineering, Key Lab of Biomedical Materials of Natural Macromolecules (Beijing University of Chemical Technology, Ministry of Education), Beijing Laboratory of Biomedical Materials, College of Materials Science and Engineering, Beijing University of Chemical Technology, Beijing, 100029, P. R. China

^b^Quzhou Institute for Innovation in Resource Chemical Engineering, Quzhou 324000, P. R. China

^c^Department of Pathology, China-Japan Friendship Hospital, Beijing 100029, PR China

^d^Department of General Surgery, China-Japan Friendship Hospital, Beijing 100029, P. R. China

^#^Both authors contributed equally to this work.

*To whom correspondence should be addressed.

Email: zhgcmd@163.com (G. Zhang), huyang@mail.buct.edu.cn (Y. Hu), xufj@mail.buct.edu.cn (F.-J. Xu).

**2. Experimental section**

***2.1. Materials***

1-(3-Dimethylaminopropyl)-3-ethylcarbodiimide hydrochloride (EDC) and N-Hydroxysuccinimide (NHS) were purchased from Energy Chemical (China). Anhydrous ethanol was purchased from Tianjin Fuyu Fine Chemical (China). Trition X-100 was purchased from Xilong Chemical (China). Prothrombin time (PT) & Activated partial thromboplastin time (APTT) kits were purchased from Heall Bio-science Technology (China). Lactate dehydrogenase (LDH) kit was purchased from Dojindo Shanghai Laboratories (China). Mouse fibroblast (L929) cell line was purchased from Chinese Academy of Medical Sciences & Peking Union Medical College (China). 3-(4, 5)-dimethylthiahiazo (-z-y1)-3, 5-di-phenytetrazoliumromide (MTT), Vitamin B2, Nitrotetrazolium blue chloride (NBT) and DL-Methionine were purchased from Energy Chemical (China). Calcein/propidium iodide Cell Viability/Cytotoxicity Assay Kit (Calcein/PI) was purchased from Beyotime Biotechnology (China). 2, 2-diphenyl-1-picrylhydrazyl (DPPH) was purchased from TCI Chemical Shanghai (China). Coomassie brilliant blue G-250 was purchased from Bioroyee Biotechnology Co., Ltd (China). The Rat Fibrinogen (FIB) Assay Kit was purchased from Nanjing Jiancheng Bioengineering Institute (China). The Reactive Oxygen Species (ROS) Assay Kit was purchased from Beyotime Biotechnology (China).

***2.2. Preparation of Z and ZC nanoassemblies***

Zein (140 mg) was dissolved in an ethanol-water solution (20 mL, at the volume concentrations of 65% or 75% or 85%) at room temperature (RT), deionized (DI) water (20 mL) was then added to the solution, transforming the clear zein solution into a milky dispersion. The nanoassemblies dispersion was subsequently freeze-dried to obtain dry samples, referred to as Z_65_ or Z_75_ or Z_85_. The ZC nanoassemblies dispersion and dry samples (ZC_65_ or ZC_75_ or ZC_85_) were prepared using the same method.

***2.3. Physical characterization***

*Fluorescence spectrophotometer assay*: Ethanol aqueous solution (at the volume concentrations of 65%) was used to prepare Z or ZC sample solution (1 mg/mL). Fluorescence spectroscopy was conducted using a fluorescence spectrophotometer (Hitachi F-7000, Japan), with an excitation wavelength of 280 nm, an emission wavelength of 300-500 nm, excitation and emission slit widths of 5 nm, and a scanning speed of 600 nm/min. A phosphate buffer was used as a blank [S1, S2].

*Fourier transform infrared (FTIR) spectroscopy:* FTIR spectra were detected at room temperature by a FTIR spectrometer Nicolet IS 10 (Thermo Scientific, USA). Z and ZC nanoassemblies were prepared as KBr pellets for the FTIR measure. The spectra of KBr pellet without sample were collected as the background spectra. Absorbance spectra from 400 to 4000 cm^-1^ were obtained at the resolution and scanning time of 4 cm^-1^ and 64 times, respectively. Secondary structures of the protein were determined by the shape of the amide I band (1600-1700 cm^-1^) using the PeakFit V4 software. The bands at 1650-1670 cm^-1^, 1600-1640 cm^-1^, 1680-1685 cm^-1^ and 1640-1650 cm^-1^ were attributed to α-helix, β-sheets, β-turns and unordered coil, respectively [S3].

*SEM observation:* To observe the morphology of Z and ZC nanoassemblies, the dry assemblies were fixed onto an appropriative platform by conductive tapes and coated with platinum, prior to SEM observation (JEOL, JSM-7500F, Japan). The particle size was calculated by analyzing SEM graph using Nano measure1.2 software (for each sample, only unsheltered particles were calculted for average diameter; n ≥ 200). The gelatin sponges (Z@GS or ZC@GS) were also fixed onto an appropriative platform coated with platinum, to expose their top-surfaces for SEM observation. To observe the interior morphologies of gelatin sponges (Z@GS or ZC@GS), the sponges were rapidly quenched in liquid nitrogen, carefully cut into two pieces, fixed onto an appropriative platform by conductive tapes and coated with platinum, prior to visualizing their longitudinal-sections by SEM (JEOL, JSM-7500F, Japan).

*Porosity test:* The porosity of gelatin sponges (GS, Z@GS, and ZC@GS) was determined by an ethanol displacement method [S3, S4]. The pre-weighed sponge sample with a calculated volume was immersed in a certain amount of anhydrous ethanol for 30 min, and then the sponge was removed and weighed again. The porosity (%) of sample was calculated from equation (1),

$\mathrm{Porosity}\left( \% \right)=\frac{m_{1}-m_{0}}{\rho V_{0}}\times100\%$ (1),

where m_0_ and m_1_ represent the weight of the sponge before and after immersion in ethanol, respectively. *V_0_* is the volume of the sponge sample and ρ is the density of ethanol (0.785 g/cm^3^). Three duplicate samples were performed for each group.

*Liquid-absorption test:* The liquid-absorption ratio was measured to compare the long-term liquid-absorption property of gelatin sponges (GS, Z@GS, and ZC@GS). The pre-weighed sponge samples were immersed in abundant liquid (PBS or citrated whole blood extracted from Sprague–Dawley (SD) rats) until the sponges were saturated with absorbent liquid. Then the sponge was removed and weighed again. The swelling ratio (%) of sample was calculated from equation (2),

$Swelling ratio \left( \% \right)=\frac{W_{1}-W_{0}}{W_{0}}\times100\%$ (2),

where w_0_ and w_1_ represent the weight of the sponge before and after immersion in abundant liquid, respectively. Three duplicate samples were performed for each group.

*Compression test*: In the compression tests, the sponges (GS, Z@GS, and ZC@GS) with cubical shape (5^3^ mm^3^) were compressed with a speed of 5 mm·min^-1^. The compression stroke is 4.5 mm, maximum load of 40 N [S5].

***2.4. In vitro hemolysis and cell viability assays***

*Hemolysis assay:* The hemolysis rate of GS, Z@GS and ZC@GS were measured using the standard procedures of hemolysis assay as described in our previous work [S3, S6]. Key procedures were provided as follows: 1) Whole blood was freshly drawn from the heart of healthy Sprague-Dawley (SD) rats (from Beijing vital river laboratory animal technology, China), stored in typical anticoagulant tube (containing 3.2% sodium citrate, which was named as citrated whole blood!!, also used in the following *in vitro* hemostatic assays) and used to prepare RBC suspension (2%, v/v and 4%, v/v, in normal saline). 2) As for GS, Z@GS or ZC@GS sponges, 5 mg of sample was placed in one 2 mL centrifuge tube and incubated with 1 mL of RBC suspension (2%, v/v) at 37℃ for 3 h. 3) Trition X-100 solution (2% in DI water) and normal saline were incubated with equal volume of RBC suspension (4%, v/v) at 37℃ for 3 h, which were set as positive and negative control groups, respectively. 4) Three duplicate samples were performed for each group.

*Cell viability assays:* The key procedures of MTT assay were provided as follows: Firstly, the leachates from GS, Z@GS, and ZC@GS sponges were prepared by the extraction of sponges with cell culture medium (named as 1640 medium, standard RPMI-1640 medium containing 1% penicillin/1% streptomycin&10% fetal bovine serum) at 37℃ for 24 h, to achieve the final sample concentration of 1 mg mL^-1^. Secondly, L929 cells were seeded into a 96-well plate at a density of 10^4^ cells/well (each well contains 100 μL of 1640 medium) and incubated for 24 h. Finally, the medium in 96-well plate was replaced with 100 μL of as-prepared sponge leachates and incubated for 24 h, prior to evaluating the relative viability of L929 cells using the standard MTT procedures (optical absorbance at 490 nm, using dimethyl sulfoxide) as described in our previous work [S3, S6]. Three duplicate samples were performed for each group.

***2.5. The free radical scavenging assays***

The DPPH scavenging assays of the GS, Z@GS, and ZC@GS samples were assessed as follows: 1) The sponges were cut into ultrasmall particles and dispersed into DPPH methanol solution (0.1 mM) at the concentration of 2 mg mL^-1^. The blank control group was the DPPH methanol solution alone. 2) All the samples were incubated in a completely dark environment for 30 min. 3) The absorbance of each sample was measured at 517 nm and the DPPH scavenging efficiency (%) was calculated from equation (3),

$\text{DPPH· (\%)=}\left( \text{1-}\frac{\text{A}_{\text{s}}}{\text{A}_{\text{0}}} \right)\text{×100\%}$ (3),

where A_0_ and A_s_ were the OD 517 values of the blank and sample groups, respectively.

The superoxide radical (O_2_·^-^) scavenging assay of the GS, Z@GS and ZC@GS samples were assessed as follows. First, the O_2_·^-^ working solution was prepared by dissolving riboflavin (20 μM), DL-methionine (12.5 mM), and nitro blue tetrazolium (75 μM) into PBS solution (25 mM, pH 7.4) in a completely dark environment. Second, the GS, Z@GS and ZC@GS were cut into ultrasmall particles and dispersed into the aforementioned working solution at the concentration of 2 mg mL^-1^. The blank control group was the working solution alone. Third, all the samples were incubated upon ultraviolet irradiation for 15 min. Finally, the absorbance of each sample was measured at 560 nm and the O_2_·^-^ scavenging efficiency (%) was calculated from equation (4),

$O_{2}\cdot^{-}\left（ \% \right）=\frac{A_{s-}A_{0}}{A_{1}-A_{0}}\times100\%$ (4),

where A_s_ and A_1_ are the OD 560 values of the sample and blank groups after irradiation, and A_0_ is the OD 560 value of the blank group before irradiation.

The key procedures of intracellular reactive oxygen scavenging assay were provided as follows: Firstly, the leachates from GS, Z@GS, and ZC@GS sponges were prepared by the extraction of sponges with cell culture medium (named as DMEM medium, standard DMEM medium containing 1% penicillin/1% streptomycin&10% fetal bovine serum) at 37℃ for 24 h, to achieve the final sample concentration of 1 mg mL-1. Secondly, L929 cells were seeded into a 12-well plate at a density of 7*10^4^ cells/well (each well contains 1 mL of DMEM medium) and incubated for 24 h. Secondly, The DMEM medium in the wells was replaced with DMEM containing 10 μg/mL lipopolysaccharide (LPS), and the cells were incubated for 24 hours. Following incubation, the culture medium was aspirated, and the wells were rinsed with PBS. The LPS-containing medium was then replaced with 1 mL of the prepared sponge filtrate, and incubation continued for an additional 24 hours. After the final incubation period, cells were washed twice with serum-free DMEM. Subsequently, 500 μL of pre-diluted DCFH-DA (diluted 1:500 in serum-free DMEM) was added to each well, and the plate was incubated in the dark for 30 mins to allow staining. Following staining, cells were washed twice with serum-free DMEM. Cellular fluorescence was observed and captured using an inverted fluorescence microscope and quantify fluorescence intensity using image J. Three duplicate samples were performed for each group.

***2.6. In vitro hemostatic assays***

*BCI assay:* The blood clotting index (BCI) assays of GS, Z@GS and ZC@GS sponges samples were performed as follows [S3]:

5 mg of GS, Z@GS or ZC@GS sponges were placed in one 1.5 mL centrifuge tube. Second, 50 μL of citrated whole blood was mixed with 5 μL of CaCl_2_ aqueous solution (0.2 M), and immediately added to the sponge pieces followed by incubation at 37°C for 65 s. Finally, 5 mL of deionized water was added to (sponge/blood mixture) to lyse uncoagulated RBCs for an additional 3 mins. The BCI values of GS, Z@GS and ZC@GS sponges were measured by quantifing the percentage of RBCs (released hemoglobin, determined by optical absorbance at 545 nm) not entrapped in the blood clots. To be noted, the dynamic BCI values of GS, Z_65_@GS or ZC_65_@GS were also evaluated for continuous time intervals (50-100 s). Three duplicate samples were performed for each group.

*PT & APTT assays:* The PT&APTT assays of GS, Z@GS, and ZC@GS sponges were performed as described in our previous work [S3]. Key procedures were provided as follows: The PT/APTT values of citrated whole blood were firstly measured as Blank group (untreated platelet-poor plasma, PPP), using the PT & APTT kits according to the supplier’s protocol. For the PT&APTT assays, 5 mg of GS, Z@GS or ZC@GS sponges was incubated with 200 μL of PPP at 37℃ for 30 mins, prior to transferring these sample-treated PPP to a new centrifuge tube and determinig the PT/APTT values according to the supplier’s protocol. PT/APTT values of these samples were normalized by the blank group (that is to be displayed as percentage of blank group). Three duplicate samples were performed for each group.

*Platelet-adhesion assays:* The platelet-adhesion assays of GS, Z@GS, and ZC@GS sponges were performed in the presence of plasma proteins (using platelet-rich plasma, PRP) as described in our previous work [S3]. Key procedures of PRP condition were provided as follows: 1) Citrated whole blood was centrifuged (150 g, 10 mins) to remove RBCs and to carefully collect the supernatant as PRP. The original PRP was diluted by equal volume of PBS (pH = 7.4) to obtain diluted PRP with a close volume with the original citrated whole blood. 2) 5 mg of sample was placed in one 1.5 mL centrifuge tube and added with 100 μL of diluted PRP at 37℃ for 5 mins. 3) After incubation, the non-adherent platelets were separated from samples by PBS washing and trasnferred to one new centrifuge tube. 4) The platelet-adhesion ratios of samples were measured by quantifing the percentage of platelets (determined by the LDH kit, according to the supplier’s protocol) adherent to sponges. Notably, the number of adherent platelets was determined by subtracting the number of pristine plateletet (in 100 μL of diluted PRP) and the number of the non-adherent platelets (in PBS washing solution after incubation & on the surface of incubated centrifuge tube), both of which were determined by LDH kit according to the supplier’s protocol. 5) Three duplicate samples were performed for each group.

*RBC-adhesion assays (diluted whole blood):* The RBC-adhesion assays of GS, Z@GS, and ZC@GS sponges were performed in the presence of plasma proteins (using diluted whole blood), adopting the modified procedures as described in our previous work [S3]. Key procedures of diluted whole blood condition were provided as follows: 1) Citrated blood was diluted with PBS (pH = 7.4) at the volume ratio of 1:19 to obtain diluted whole blood (5%, v/v). 2) 5 mg of sample was placed in one 2 mL centrifuge tube and added with 100 μL of diluted whole blood at 37℃ for 30 mins. 3) After incubation, the non-adherent RBCs were separated from samples by PBS washing and trasnferred to one new centrifuge tube. 4) The RBC-adhesion ratios of samples were measured by quantifing the percentage of RBCs (intracellular hemoglobin, determined by optical absorbance at 520 nm) adherent to sponges. Notably, the number of adherent RBCs was determined by subtracting the number of pristine RBCs (in diluted whole blood) and the number of the non-adherent RBCs (in new tube), both of which were directly related to the concentration/absorbance after being diluted to the equal volume by PBS. 5) Three duplicate samples were performed for each group.

*Fgn-adsorption assay:* Key procedures of PPP condition were provided as follows: 1) Citrated blood was centrifuged (3000 rpm, 15 mins) to carefully collect the supernatant as platelet-poor plasma (PPP). 2) 5 mg of GS (or Z@GS or ZC@GS) was placed in one 2-mLcentrifuge tube (tube one) and incubated with 25 μL of PPP at 37℃ for 5 mins. 3) 400 μL of PBS (pH = 7.4) was added to tube one, prior to transferring free PPP to a new centrifuge tube (tube two), then the tube two was filled to 2 mL. 4) Draw a standard curve using the Fgn reagent kit and perform subsequent tests according to the instructions. 5) Finally, the solution was measured by excitation at wavelength of 450 nm with the Multiwall Plate Reader (BioTek Cytation 3, USA). 6) Finally, the Fgn-adsorption ratio was calculated from equation (5),

$\eta\left( Fgn-adsorption ratio \right)=\left( 1-\frac{C_{s}}{C_{b}} \right)\times100\%$ (5),

Where C_s_ and C_b_ are the OD 450 values of the above sample and control group.

*Whole-protein-adsorption assay*: Key procedures of PPP condition were provided as follows [S6, S7]: 1) Citrated blood was centrifuged (3000 rpm, 15 mins) to carefully collect the supernatant as platelet-poor plasma (PPP). 2) 5 mg of GS (or Z@GS or ZC@GS) was placed in one 2-mL centrifuge tube (tube one) and incubated with 25 μL of PPP at 37℃ for 5 mins. 3) 400 μL of PBS (pH =7.4) was added to tube one, prior to transferring free PPP to a new centrifuge tube (tube two), then the tube two was filled to 2 mL. 4) 20 μL liquid was transferred to a 96-well plate, and then 200 μL coomassie brilliant blue G-250 was added to react for 5 mins. 5) Finally, the solution was measured by excitation at wavelength of 595 nm with the Multiwall Plate Reader. Finally, the whole-protein-adsorption ratio was calculated from equation (6),

$\eta\left( whole-protein-adsorption ratio \right)=\left( 1-\frac{C_{s}}{C_{b}} \right)\times100\%$ (6),

Where C_s_ and C_b_ are the OD 595 values of the above sample and control group.

To be noted, the whole-protein-adsorption of gelatin sponges was also performed under PPP condition. The PPP (at the concentration of 200/160/120/80/60/40/20 μg/mL, respectively) was prepared to drawing standard curve.

*In vivo study in artery-injury models adopting healthy rats:* Key procedures of femoral-artery-injury model in healthy rats were provided as follows [S3]: 1) SD rats (male, 160-190 g, from Beijing vital river laboratory animal technology, China) were randomly divided into three groups: GS, Z_65_@GS, and ZC_65_@GS group were all treated with 2 × 2 cm of sponge. 2) After anesthetization, the femoral artery and surrounding vein/nerve of rats were cut off with a scalpel together to create the femoral-artery-injury model, since the latter two were difficult to be separated from the artery. 3) The cut-off femoral artery of each group was allowed for free bleeding for 15 s prior to applying the sponge samples, with the blood loss collected counted for the pre-treatment blood loss. 4) The sponge samples of each group were applied onto the injury site and immediately covered by a weigh (100  g) for standard pressure. The sponge samples were kept on the wound for the first 2 mins and the subsequent every min (i.e. the bleeding time of three groups was recorded as 2, 3, 4, 5, ... mins), prior to measuring post-treatment blood loss. 5) Only rats with the pre-treatment blood loss of 260-420 mg (denoted as rats with normal blood loss) were selected for the final analysis of the post-treatment blood loss and bleeding time, to reduce the influence of individual differences of the rats and operation error among the three groups (for GS, Z_65_@GS and ZC_65_@GS groups, n ≥ 3).

*In vivo study in liver-injury models adopting healthy rats:* Key procedures of liver-injury model in healthy rats were provided as follows: 1) SD rats (male, 160-190 g, from Beijing vital river laboratory animal technology, China) were randomly divided into three groups: GS, Z_65_@GS, and ZC_65_@GS group were all treated with 2 × 2 cm of sponge. 2) After anesthetization, the rat abdomen was incised to expose the liver. The liver was gently lifted, and surface moisture was carefully wiped from its surface. Pre-weighed filter paper was placed beneath the liver. A circular wound surface measuring Φ5×2 mm was created in the left upper lobe of the liver using a sterilized biopsy punch, thus establishing the liver injury model. 3) The liver wound of each group was allowed for free bleeding for 10 s prior to applying the sponge samples, with the blood loss collected counted for the pre-treatment blood loss. 4) The sponge samples of each group were applied onto the injury site and the subsequent every 30 s prior to measuring post-treatment blood loss. 5) Only rats with the pre-treatment blood loss of 50-150 mg (denoted as rats with normal blood loss) were selected for the final analysis of the post-treatment blood loss and bleeding time, to reduce the influence of individual differences of the rats and operation error among the three groups (for GS, Z_65_@GS and ZC_65_@GS groups, n ≥ 3).

*In vivo study in coagulopathic artery-injury model (adopting heparinized rats):* The key procedures for establishing the heparinized rat femoral-artery-injury model are as follows: 1) SD rats (male, 160-190 g, from Beijing vital river laboratory animal technology, China) were anesthetized and administered sodium heparin (100 U/kg) through tail vein injection [S3]. After a circulation period of 10 mins, the SD rats were designated for subsequent assays as heparinized models. 2) Whole blood was freshly drawn from the hearts of heparinized rats and stored in anticoagulant tubes (containing 3.2% sodium citrate). Blood samples from three rats were used for APTT and PT assays (using the PT & APTT kits according to the supplier’s protocol) to compare with those of healthy rats and confirm the successful establishment of the coagulopathic rat model. 3) Notably, the whole blood sample from one heparinized rat was used to evaluate the *in vitro* hemostatic properties of GS and ZC_65_@GS sponges under coagulopathic conditions (i.e. 5 mg of samples were incubated with 50 μL of citrated whole blood & 5 μL of CaCl_2_ for 10 mins), following the same procedures described for the blood clotting index (BCI) assay. 4) Immediately after the 10-mins circulation period, the femoral artery injury model was established in heparinized rats to assess the hemostatic performance of GS and ZC_65_@GS sponges under coagulopathic conditions *in vivo*, using the same methodology as for healthy rats. It is important to note that pre-treatment blood loss ranged from 350 to 550 mg, and three replicates were performed for each group (n = 3).


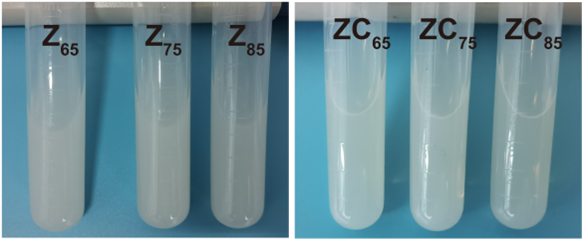


**Fig. S1.** Photographs of dispersions of Z_65_, Z_75_ Z_85_, ZC_65_, ZC_75_, and ZC_85_ at varied ethanol/water content (65%, 75%, and 85%).

**Table S1.** Characterization of the element content of Z and ZC

|  | C% | N% | H% | S% | grafting rate (%) |
| --- | --- | --- | --- | --- | --- |
| Z | 52.51 | 15.42 | 6.97 | 0.59 | - |
| ZC | 52.21 | 14.57 | 0.48 | 0.48 | 6.03 |

Analysis. The mass content (x) of CA in ZC can be calculated from the following equation (1),

$\frac{N_{\mathrm{ZC}}}{C_{\mathrm{ZC}}}=\frac{N_{Z}\times(1-x)}{C_{Z}\times\left( 1-x \right)+C_{\mathrm{CA}}\times x}$ (1),


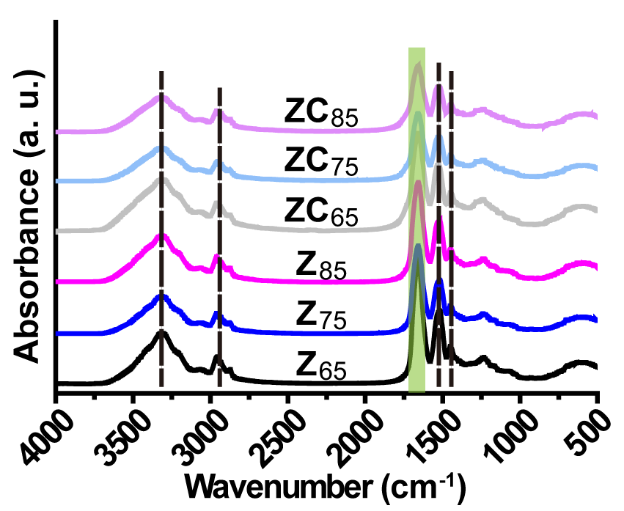


**Fig. S2.** FTIR spectra of Z and ZC nanoassemblies.

**Analysis.** The band corresponding to the stretching of the N-H and O-H bonds of the amino acids of the protein appears 3307 cm^-1^. Band appears at 1660 cm^-1^, corresponding to stretching of the carbonyl (C=O) of amide groups belonging to the peptide groups (amide I, 1600-1700 cm^-1^). The band at 1535 cm^-1^ is called amide II and corresponds to the angular deformation vibrations of the N-H bond, the band at 1230 cm^-1^ corresponds to the axial deformation vibrations of the C-N bond. Deconvolution and curve fitting of the spectrum of the amide I band (1600–1700 cm^-1^) were performed to determine the secondary structure content of Z and ZC nanoassemblies.


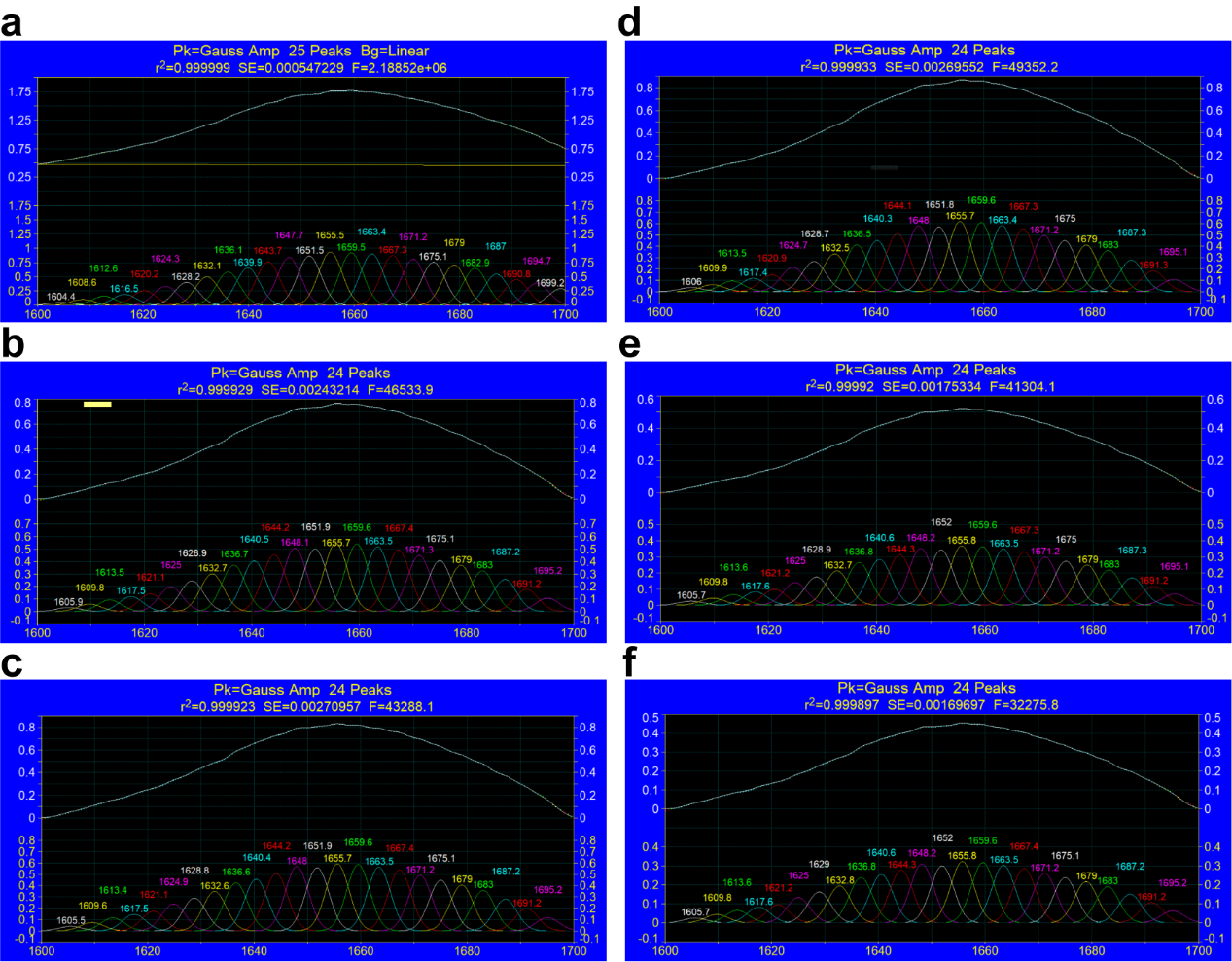


**Fig. S3.** Reconstituted spectra after curve fitting of FTIR spectra (at 1600–1700 cm^-1^) of (a) Z_65_, (b) Z_75_, (c) Z_85_, (d) ZC_65_, (e) ZC_75_ and (f) ZC_85._

**Analysis.** Taking the reconstituted spectra of Z_65_ as an example to calculate the content of secondary structure. As shown in Fig. S3a, after the curve fitting of FTIR spectra, the reconstructed spectra of β-sheets (1600-1640 cm^-1^) are divided into peaks of 1604.4, 1608.6, 1612.6, 1616.5, 1620.2, 1624.3, 1628.2, 1632.1 and 1636.1^-1^, while the corresponding areas of peaks are 0.284, 0.569, 0.885, 1.068, 1.422, 1.857, 2.242, 2.782 and 3.255, respectively. Thus, the total area occupied by β-sheets (1600-1640 cm^-1^) is 21.385. Similarly, the total area occupied by α-helix (1650-1670 cm^-1^), β-turns (1680-1685 cm^-1^) and unordered coil (1640-1650 cm^-1^) is calculated to be 29.582, 10.632 and 12.591, respectively. Thus, the total area of four types of secondary structure is 67.169 (as the sum of 14.364, 29.582, 10.632 and 12.591). Finally, the content of β-sheets was obtained by the ratio of 14.364 and 67.169.


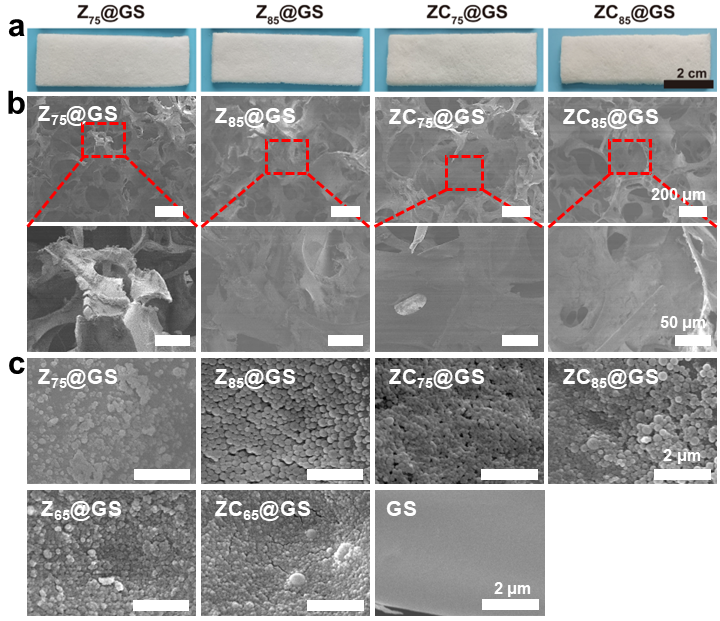


**Fig. S4.** (a) Photograph, (b)SEM images of the top-surface view of Z_75_@GS, Z_85_@GS ZC_75_@GS and ZC_85_@GS. (c) SEM images of the top-surface view of GS, Z_65_@GS, Z_75_@GS, Z_85_@GS, ZC_65_@GS, ZC_75_@GS and ZC_85_@GS (at high magnification).


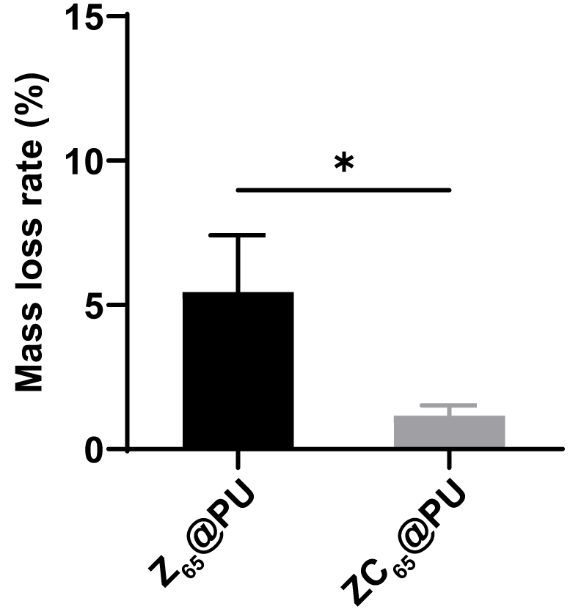


**Fig. S5.** Stability test of Z_65_@PU and ZC_65_@PU coatings in normal saline (data are presented as the mean ± SD, n = 3, Student's t-tests). (ns denotes no significant difference, while * p < 0.05, **p < 0.01, and ***p < 0.001 represent statistically significant differences).

**Analysis.** Soak Z_65_@PU and ZC_65_@PU in normal saline for weight loss analysis to reflect Z (or ZC) coating stability. After soaking Z_65_@PU and ZC_65_@PU in normal saline for 12 hours, the quality loss rate of Z coating was 5.4%, while the quality loss rate of ZC coating was only 1.2% (Fig. S5). This indicates that the stability of the Z coating is relatively high, possibly due to the hydrophobic properties of Z assemblies and the formation of intermolecular interactions with the substrate surface. Compared to Z coating, ZC coating has higher stability, which may be due to the phenolic hydroxyl groups in ZC providing good adhesion ability (hydrogen bonding, π interactions, etc.)


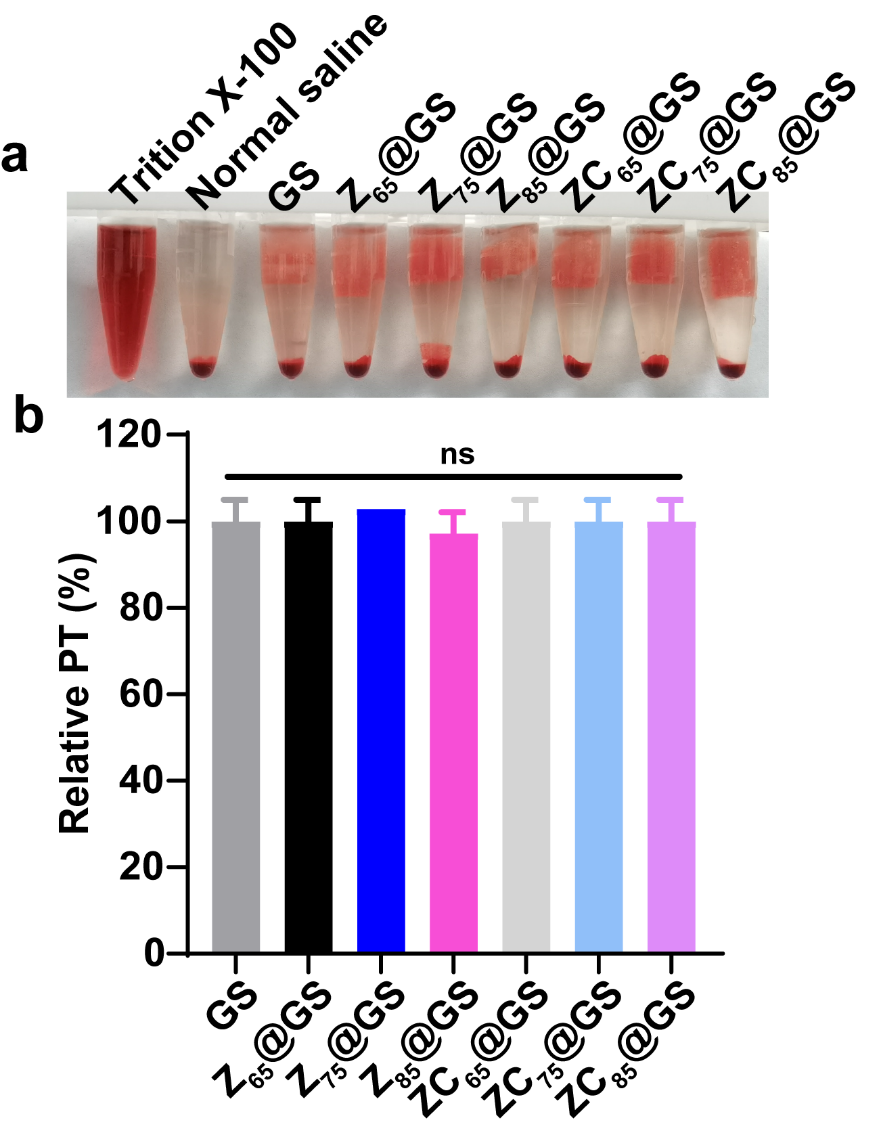


**Fig. S6.** (a) Photographs of RBCs treated with normal saline, Trition X-100, GS, Z@GS, and ZC@GS. (b) PT of GS, Z@GS and ZC@GS (data are presented as the mean ± SD, n = 3, one-way ANOVA).


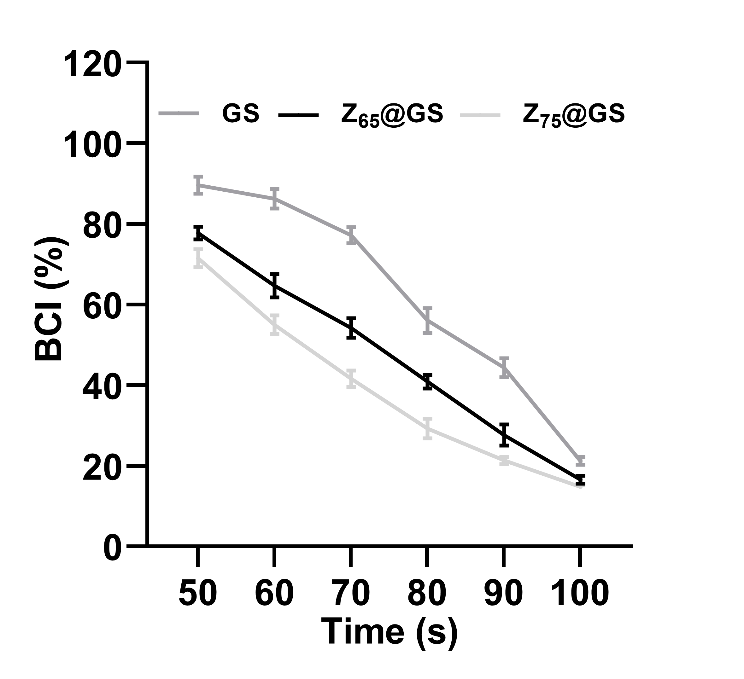


**Fig. S7.** BCI values of GS, Z_65_@GS, and ZC_65_@GS with different incubation time. (data are presented as the mean ± SD, n = 3, one-way ANOVA)


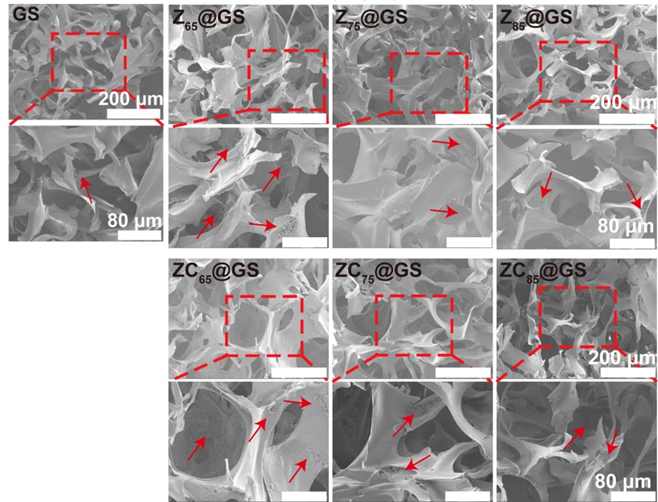


**Fig. S8.** Representative SEM images of adherent RBCs (with plasma proteins) on GS, Z@GS, and ZC@GS.


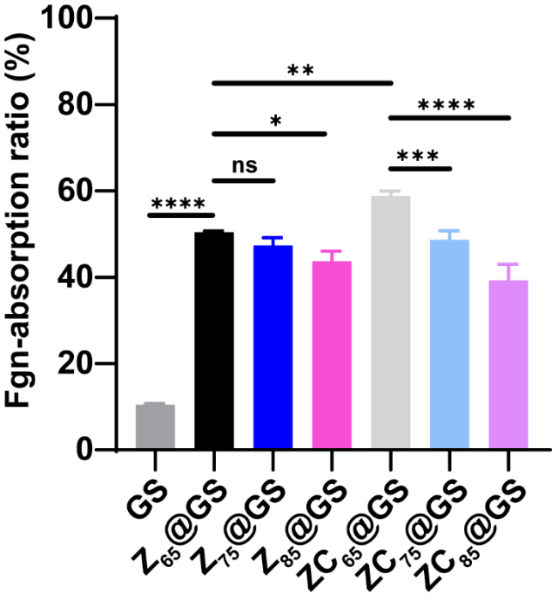


**Fig. S9.** Fgn-absorption ratio under PPP conditions of GS, Z@GS and ZC@GS (data are presented as the mean ± SD, n = 3, one-way ANOVA).


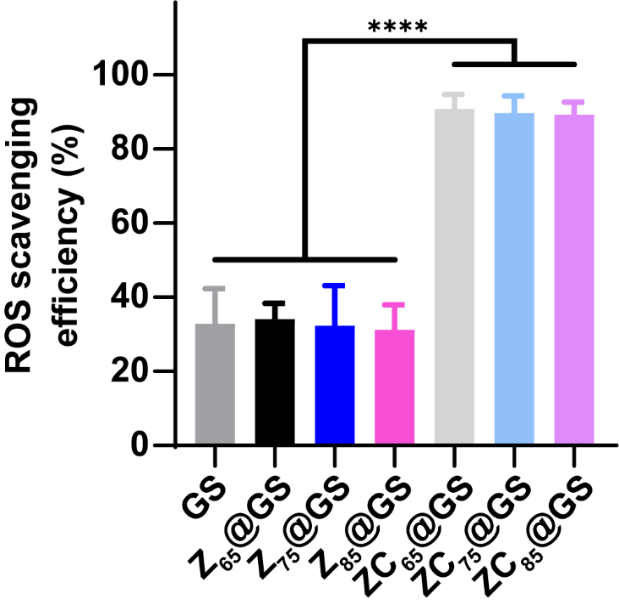


**Fig. S10.** The ROS scavenging efficiency of GS, Z@GS and ZC@GS (data are presented as the mean ± SD, n = 3, one-way ANOVA)


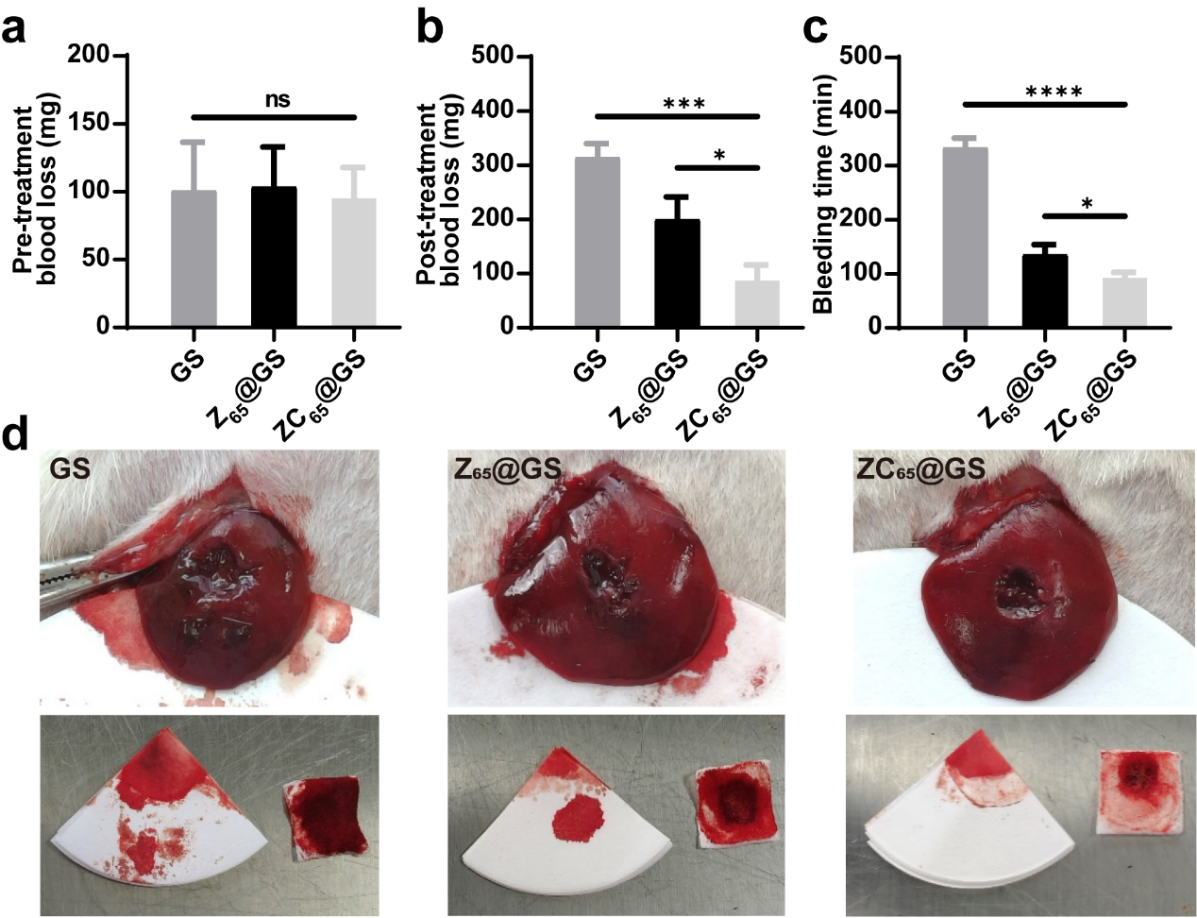


**Fig. S11.** (a) Pre-treatment blood loss (data are presented as the mean ± SD, n ≥ 3, one-way ANOVA), (b) post-treatment blood loss (data are presented as the mean ± SD, n ≥ 3, one-way ANOVA), (c) bleeding time (data are presented as the mean ± SD, n ≥ 3, one-way ANOVA) and (d) representative photographs of GS, Z_65_@GS and ZC_65_@GS in a rat liver-injury model. (ns denotes no significant difference, while *p < 0.05, **p < 0.01, and ***p < 0.001 represent statistically significant differences, one-way ANOVA).


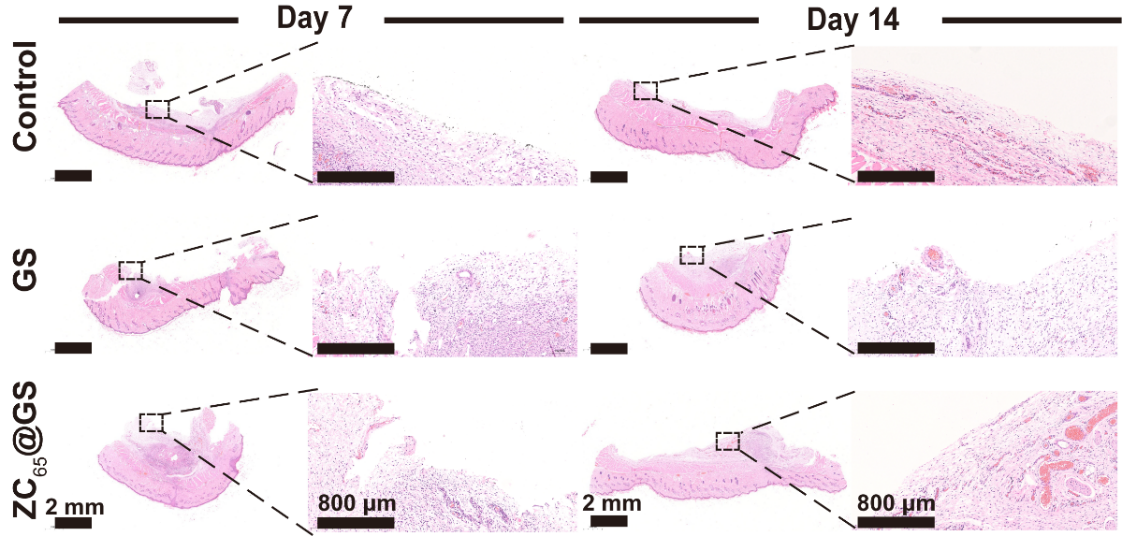


**Fig. S12.** *In vivo* H&E-stained slices of GS and ZC_65_@GS in a subcutaneous implantation model of healthy rats.


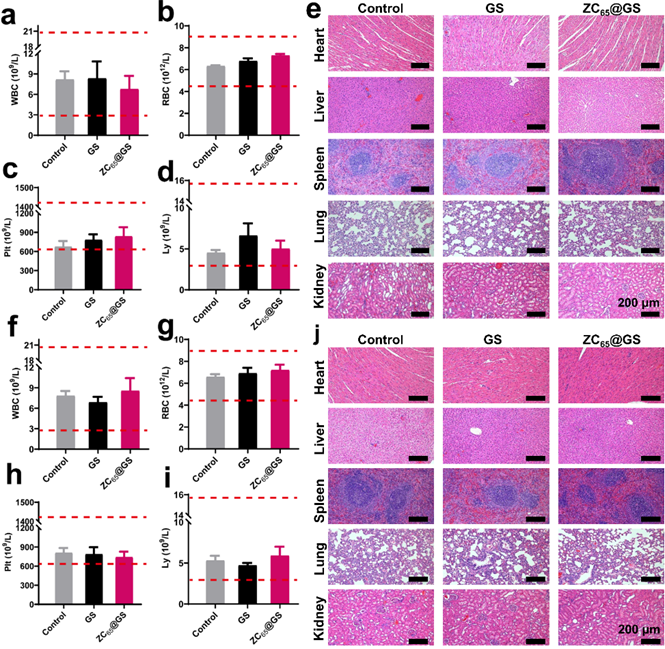


**Fig. S13.** Changing tendencies in blood phase treated with GS and ZC_65_@GS on day 7 (a-e) and 14 (f-j): (a) and (f) numbers of leukocytes; (b) and (g) number of red blood cells; (c) and (h) number of blood platelet; (d) and (i) numbers of lymphocytes; (e) and (j) H&E staining images of heart, liver, spleen, lung and kidney treated with GS and ZC_65_@GS. The dotted range is the normal range of each indicator. (data are presented as the mean ± SD, n = 3).

**References**

1. S. Yan, J. Xu, S. Zhang, Y. Li, Effects of flexibility and surface hydrophobicity on emulsifying properties: Ultrasound-treated soybean protein isolate, LWT--Food Sci. Technol. 142 (2021) 110881.
2. S. Yan, Y. Yao, X. Xie, S. Zhang, Y. Huang, H. Zhu, Y. Li, B. Qi, Comparison of the physical stabilities and oxidation of lipids and proteins in natural and polyphenol-modified soybean protein isolate-stabilized emulsions, Food Res. Int. 162 (2022) 112066.
3. Y. Wang, J. Lin, H. Fu, B. Yu, G. Zhang, Y. Hu, F.-J. Xu, A Janus gelatin sponge with a procoagulant nanoparticle-embedded surface for coagulopathic hemostasis, ACS Appl. Mater. Interfaces 16(1) (2024) 353-363.
4. T.M. Tamer, M.H. Alsehli, A.M. Omer, T.H. Afifi, M.M. Sabet, M.S. Mohy-Eldin, M.A. Hassan, Development of polyvinyl alcohol/kaolin sponges stimulated by marjoram as hemostatic, antibacterial, and antioxidant dressings for wound healing promotion, Int. J. Mol. Sci., 22(23) (2021) 13050.
5. C. Ding, K. Cheng, Y. Wang, Y. Yi, X. Chen, J. Li, K. Liang, M. Zhang, Dual green hemostatic sponges constructed by collagen fibers disintegrated from Halocynthia roretzi by a shortcut method, Mater. Today Bio 24 (2024) 100946.
6. Y. Su, M. Niu, K. Xu, C. Xu, P. Yang, Y. Hu, F.-J. Xu, Cationic starch microparticles with integrated antibacterial and hemostatic performance, Sci. China: Technol. Sci 67(10) (2024) 3235-3246.
7. Y. Su, H. Chen, Q. Liu, X. Ding, R. Lian, Y. Hu, F.-J. Xu, Thermoresponsive gels with embedded starch microspheres for optimized antibacterial and hemostatic properties, ACS Appl. Mater. Interfaces 16(10) (2024) 12321-12331.
8. Y.-B. Zhang, H. J. Wang, A. Raza, C. Liu, J. Yu, J. Y. Wang, Preparation and evaluation of chitosan/polyvinylpyrrolidone/zein composite hemostatic sponges, Int. J. Biol. Macromol. 205 (2022) 110-117.
9. A. Raza, Y. Zhang, U. Hayat, C. Liu, J.-L. Song, N. Shen, Y. Chao, H. J. Wang, J. Y. Wang, Injectable zein gel with in situ self-assembly as hemostatic material, Biomater. Adv. 145 (2023) 213225.
